# Impact of Land-Use Change on Vascular Epiphytes: A Review

**DOI:** 10.3390/plants14081188

**Published:** 2025-04-11

**Authors:** Thorsten Krömer, Helena J. R. Einzmann, Glenda Mendieta-Leiva, Gerhard Zotz

**Affiliations:** 1 Centro de Investigaciones Tropicales, Universidad Veracruzana, Xalapa 91000, Mexico; 2 Institute of Biology and Environmental Sciences, Functional Ecology, Carl von Ossietzky Universität Oldenburg, D-26111 Oldenburg, Germany; helena.einzmann@uni-oldenburg.de (H.J.R.E.); gerhard.zotz@uni-oldenburg.de (G.Z.); 3 Faculty of Biology, Philipps University of Marburg, D-35037 Marburg, Germany; glendamendieta@gmail.com; 4 Plant Ecology Division-Centro de Ornitología y Biodiversidad (CORBIDI), Lima 00051, Peru; 5 Smithsonian Tropical Research Institute, Balboa, Ancon, Panama City 0843-03092, Panama

**Keywords:** deforestation, disturbed forests, fragmentation, human disturbance, isolated remnant trees, literature search, tree plantations, secondary forests, selective logging, urban vegetation

## Abstract

Human-caused habitat conversion, degradation, and climate change threaten global biodiversity, particularly in tropical forests where vascular epiphytes—non-parasitic plants growing on other plants—may be especially vulnerable. Epiphytes play vital ecological roles, in nutrient cycling and by providing habitat, but are disproportionately affected by land-use changes due to their reliance on host trees and specific microclimatic conditions. While tree species in secondary forests recover relatively quickly, epiphyte recolonization is slower, especially in humid montane regions, where species richness may decline by up to 96% compared to primary or old-growth forests. A review of nearly 300 pertinent studies has revealed a geographic bias toward the Neotropics, with limited research from tropical Asia, Africa, and temperate regions. The studies can be grouped into four main areas: 1. trade, use and conservation, 2. ecological effects of climate and land-use change, 3. diversity in human-modified habitats, and 4. responses to disturbance. In agricultural and timber plantations, particularly those using exotic species like pine and eucalyptus, epiphyte diversity is significantly reduced. In contrast, most native tree species and shade-grown agroforestry systems support higher species richness. Traditional polycultures with dense canopy cover maintain up to 88% of epiphyte diversity, while intensive management practices, such as epiphyte removal in coffee and cacao plantations, cause substantial biodiversity losses. Conservation strategies should prioritize preserving old-growth forests, maintaining forest fragments, and minimizing intensive land management. Active restoration, including the translocation of fallen epiphytes and planting vegetation nuclei, is more effective than passive approaches. Future research should include long-term monitoring to understand epiphyte dynamics and assess the broader impacts of epiphyte loss on biodiversity and ecosystem functioning.

## 1. Introduction

Human activities, especially the conversion, degradation, and fragmentation of habitats, as well as climate change, are major threats to global biodiversity, especially in the tropics [1,2]. Habitat loss, either caused by ongoing deforestation or changes in land-use, is the largest single driver of the decline in terrestrial biodiversity [1,3]. The magnitude and negative impact of the human disturbance on tropical forests and their biodiversity are well documented (e.g., [4,5,6]). In recent decades, increases in agricultural demand, logging activities and urban growth have led to unprecedented losses of tropical forests, with annual deforestation rates of around 0.5% since the 1990s, resulting in an annual loss of tropical forests of about 7.8 million hectares until the 2000s [7], and an annual loss of 6.4 million hectares between 2010 and 2015 [8]. At present, few truly undisturbed tropical forests exist, whereas degraded and regrowing forests after the abandonment of agricultural lands are rapidly expanding, currently comprising about half of the world′s tropical forests [9,10]. Secondary forests may therefore constitute an important role as reservoirs for biodiversity [11,12]; however, the majority of relevant vegetation studies focuses on trees (e.g., [13,14]).

A smaller portion of studies are on lianas (woody vines; e.g., [15,16]), a structurally dependent component of the vegetation, which seems to be rather favored by disturbance, i.e., liana density and diversity peak following disturbance at local and forest scales [17,18]. This highlights how different components of the forests respond differently to disturbance, but research on other vegetative components of biodiversity is still very limited. For instance, despite the large-scale clearing of tropical forests since the 1970s, it took until the mid-1990s for Turner et al. [19] to show in a study from Singapore that epiphytic species, as a component of diversity, appear to be particularly threatened by habitat loss. While the species richness of the most studied life forms, such as trees (19%), shrubs (34%), herbs (17%), and climbers (23%), was much less affected, epiphyte species richness exceeded 60% loss.

Vascular epiphytes, plants that grow on other plants [20], are a conspicuous and integral component of tropical forests regarding local, regional, and continental plant diversity [21,22,23]. This very heterogeneous and taxonomically diverse group of plants contributes substantially to plant species richness, accounting for roughly 10% of all vascular plant species globally [24] and for up to 50% locally [25]. Epiphytes fulfil important ecosystem functions related to forest nutrients and water cycling [26,27,28,29]. Moreover, they provide food and habitat for canopy-dwelling fauna such as arthropods, birds or bats [30,31,32,33]. Because of these ecological functions, epiphytes are considered secondary foundation species [34]. These are species that provide structurally complex biogenic habitats that alter environmental conditions within the matrix of the primary foundation species, e.g., in this case, the host trees or shrubs [34]. Consequently, vascular epiphytes add structural complexity to forest canopies [20]. Therefore, any adverse effects on epiphytes cannot be viewed in isolation, as land-use change effects on epiphytes would have cascading consequences and similarly affect other inhabitants, such as associated fauna [34,35].

Without root contact to ground soil and dependent on the stability of their host, epiphytes might differ substantially from ground-rooted plants regarding their response to human-driven degradation and the conversion of tropical forests for agriculture and other land-use purposes. For instance, tree species richness increases very rapidly during secondary succession in Neotropical forests, with 80% recovery of old-growth values after only 20 years [36]. Moreover, in many secondary forests, tree species richness has surpassed that of old-growth forests [15], while epiphytes as canopy-dwelling organisms are specifically threatened by disturbance and deforestation [19,37,38], and their diversity is generally low in secondary forests [39,40,41].

However, little is known about how many and which epiphyte species (genera and families) may be prone to extinction or are lost, which depends on the type and magnitude of disturbance and the type of vegetation that replaces the original forest [42]. Several earlier studies (e.g., [39,40,43]) showed that compared with old-growth forests, epiphyte diversity tends to be markedly reduced following disturbance in most of the investigated human-modified habitats, but these studies were mostly carried out in humid lowland or montane forests. A growing number of later studies revealed exceptions to this trend, indicating that changes in epiphyte diversity and species composition vary significantly with the location and climate regime (e.g., rainfall and seasonality) of the study site, as well as the type and degree of habitat transformation (e.g., [41,44,45,46]). Diverging patterns, however, are actually unsurprising considering that disturbed and secondary habitats differ greatly in age and structure, and in the degree of structural differences from the original vegetation [47]. Considering this mixed picture, it is important to determine if epiphytes as a group really are particularly threatened by land-use change [48].

In this review, we summarize our current knowledge and try to find general patterns of the effects of land-use changes on epiphytes by analyzing all available information gathered through an intensive literature revision. As a basis for future work, we identify open questions within the larger context of human disturbance activities and conservation strategies for epiphytes in general. By recognizing the main aspects of research, we want to achieve the following: 1. Weigh into the mixed picture of the subject to determine if epiphytes as a group really are particularly threatened by land-use change. 2. Suggest plausible future directions of the field, identifying open questions in the larger context of land-use change. 3. Provide suggestions for possible conservation strategies for vascular epiphytes.

## 2. Materials and Methods

### 2.1. Literature Search

To gather relevant information on the impact of land-use change on epiphyte diversity and related topics, we conducted an extensive literature search for research articles, reviews, book chapters, and grey literature (e.g., theses, notes) with a publication date up to October 2024 utilizing common scientific databases including Clarivate Web of Science and the Scientific Electronic Library Online (SciELO) and internet search engines such as Google Scholar, using operators such as ‘epiphyt*’ AND ‘land-use’ OR ‘disturbance’ OR ‘deforestation’ OR ‘logging’ OR ‘remnant tree’ OR ‘plantation’ OR ‘urban’ OR ‘fragmentation’ OR ‘edge effect’ OR ‘succession’ OR ‘recolonization’ OR ‘harvest’ OR ‘trade’ OR ‘translocation’ in the titles, abstracts, and keywords. In addition, we revised the comprehensive vascular epiphyte literature database of Gerhard Zotz, which includes ca. 12,000 references, many of which are not included in the other databases. After carefully revising the abstracts of all references obtained, we excluded all publications not dealing with the study topics (e.g., focused on non-vascular epiphytes or epiphytic algae on seagrasses). We finally identified 285 relevant references in English, Portuguese, and Spanish (EndNote and Excel database; Supplementary Material, Table S1), mostly journal articles (254), but also several book chapters (19) and theses (14). A total of 156 publications focused on vascular epiphytes in general (some including hemiepiphytes, nomadic vines or accidental epiphytes; [49,50,51]), whereas 58 studies focused on orchids, 41 on bromeliads, and 30 on pteridophytes. Some of the latter research considered two of these three groups and a few also included terrestrial or saxicolous taxa.

### 2.2. Identifying the Main Aspects of Research

To obtain the main areas of research when evaluating the impact of land-use change on vascular epiphytes, we carried out a bibliometric analysis on the current state of knowledge about the impact of land-use change on vascular epiphytes. We used a text mining approach with a cooperative cluster-ordination and k-means to create groups representing the most consistent subjects [52] on the abstracts of the 285 references considered in this review. The process involved exporting references from EndNote^®^ TM 21.5 to Microsoft Word^®^. The 285 references in EndNote were exported (annotated to include the title and abstract) as an RTF extension and organized in Microsoft Word. The structure was homogenized so that each paragraph contained the title and the abstract. Spanish or Portuguese content was translated into English using DeepL translator version 24.11.4.144242. After translating and homogenizing the abstracts, the RTF file was read into R programming language [53], where we carried out cleaning and stemming procedures, to finally proceed with the analyses (ordination and clustering techniques). The abstract list was cleaned by eliminating “stop words” (such as connectors and abbreviations) and special characters (such as punctuation, numbers, and symbols) and homogenized by stemming words (e.g., removing or reducing derived words, epiphytism --> epiphyt).

Following the cleaning and stemming procedures, a word matrix was created, with 285 rows representing the publications and 4080 columns representing the stemmed words. The cells of the word matrix represented the frequency with which each word appeared in the abstract of each publication. Among the most frequent terms we found two that referred to the same concept, which were therefore combined, as follows: “epiphyte” was combined with “holoepiphyte” and “host” with “phorophyte” and “tree”. The packages qdap [54], stringr [55], striprtf [56], tm [57], tidyverse [58], and data.table [59] were used for text cleaning and stemming. The resulting word matrix was used in a detrended correspondence analysis (DCA; [60]) using the “decorana” command from the vegan package [61] to down-weigh uncommon occurrences (of words), thus lessening the influence of rare “terms” (words). This ordination technique is used to obtain a low-dimensional representation of the word matrix and reduce dimensionality (2–3 axes generally).

**Figure 1:**
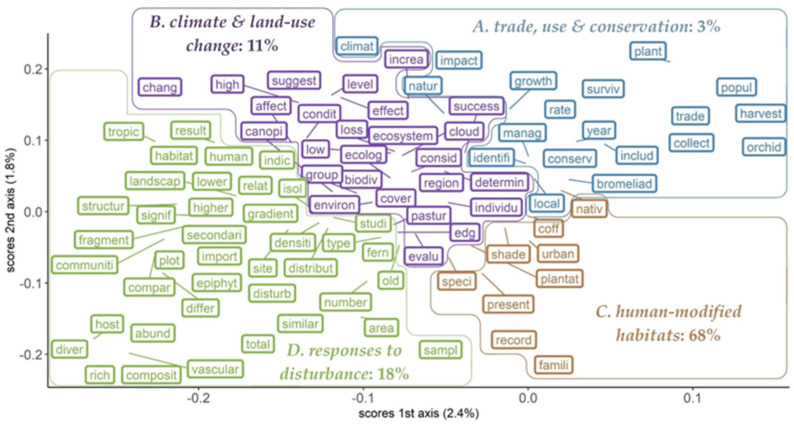
Four aspects of the research. A: *Trade, use and conservation*. B: *Climate and land-use change*. C: *Human-modified habitats*. D: *Responses to disturbance*. Here, we show the most frequent words per aspect (cluster) in relation to their frequency (those with a frequency larger than the highest 3rd quartile frequency of all clusters). The percentages of abstracts assigned to each cluster are shown (next to the cluster name). For the sake of comparison, we include a similar graph including the most common 20 words per cluster (Supplementary Material, Figure S2, the limit in words was set for visibility’s sake). Words varied in their frequency per cluster, i.e., some clusters were more consistent than others (Supplementary Material, Figure S3).

Then, to identify homogeneous groups (clusters) within the word matrix, we applied k-means clustering to the ordination scores. To find the best compromise between a low number of clusters and a high variance of the word matrix, we use the command “fviz” from the package NbClust [62]. This method identifies groups by minimizing within-cluster variation by computing the sum of all within-cluster squares (wss) for each run, which it does for a total of 10 self-defined clusters [63]. The visual assessment of the “wws” as a function of clusters was used to determine the number of suitable clusters; we also performed the same analysis using a different clustering technique (the average silhouette method), and the results were comparable (Supplementary Material, Figure S1). Once the clusters were derived from the word matrix, we assigned each abstract to one of the four resulting clusters; this involved tallying the number of words in the abstracts matching with the words defining each cluster, and once we had identified the cluster with the largest number of words matched to an abstract, this publication was assigned to the cluster (Figure 1; Supplementary Material, Table S1, list of the papers assigned to each group). We found the abstracts were grouped into four clusters representing the most common research carried out in the last two decades (Figure 1). These were plotted using the command “geom_label_repel”, which allows the text labels to shift and reconfigure away from the data points for readability, but indicates with lines where the exact point was, using the packages ggrepel version 0.9.6 and ggplot2 version 3.5.1 [64,65,66].

## 3. Results and Discussion

### 3.1. General Results

Over the last two decades, many relevant studies related to the effects of land-use changes on vascular epiphytes have been conducted, although mainly at a local scale and with a strong bias towards the Neotropics compared to tropical Africa and Asia, or temperate regions [20]. Only a few publications give a broader overview [20,35,42,67,68] but do not cover all related topics in detail.

**Figure 2:**
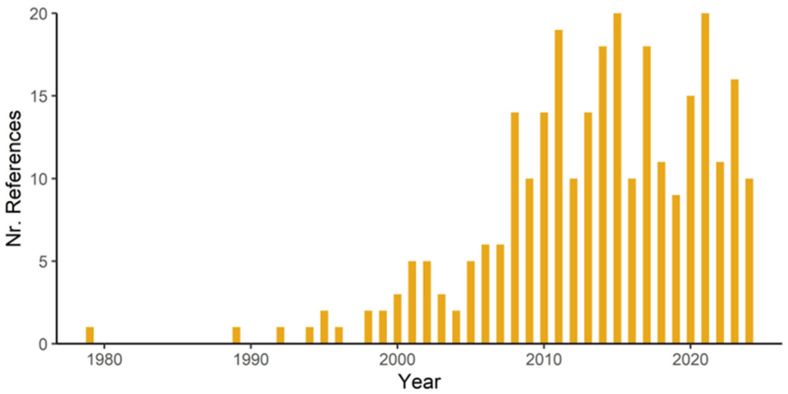
The number of references considering the effect of land-use changes on epiphytes per year.

The first study considering the effects of land-use changes on epiphytes was published in Selbyana by Michael Madison in 1979 [69], addressing the distribution of epiphytes in a rubber plantation in Malaysia (Figure 2). During the following two decades, only 10 additional studies were published. This number increased in the 2000s to 59 and reached 143 in the 2010s, while during the last five years, the annual number of papers ranged between 10 and 20, with a yearly mean of 14.4 published studies.

**Figure 3:**
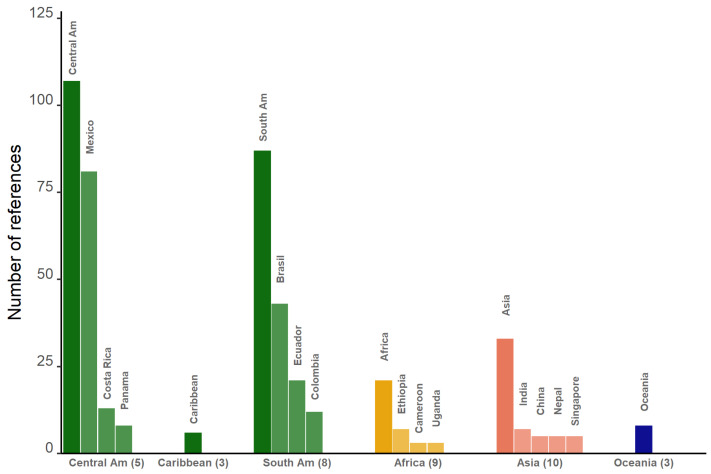
The number of articles/studies per major region/continent (first bars; indicating the number of countries in brackets on the x-axis) and main countries (following bars).

The major regions/continents and countries where these studies were conducted (Figure 3) show a strong geographic bias towards Central America (107 papers). In Mexico alone 81 studies were conducted, slightly less than those conducted in eight countries of South America (87), of which in turn almost half were situated in Brazil (43). In ten countries of Asia, 33 studies were conducted, led by India (7), and in nine countries of Africa, 21 studies were conducted, led by Ethiopia (7). In three Caribbean countries, six studies were conducted, and eight were conducted in Oceania. Furthermore, there are 23 studies, mainly review papers, with no clear geographic focus.

### 3.2. Identifying the Main Aspects of Research

The text-mining analysis based on almost 300 abstracts (summing up 4080 words) resulted in four clusters (Figure 1; Supplementary Material, Figures S1 and S2), which we classified into (A) *trade, use and conservation*, (B) ecological effects of *climate and land-use change*, (C) diversity across *human-modified habitats*, and (D) ecological effects of *responses to disturbance*. This classification was based on the most frequent words grouped per cluster and the majority of abstract titles contained in each cluster in the ordination. The clusters grouped a very uneven number of abstracts, with the largest number indicating that most research focuses on evaluating epiphyte diversity across *human-modified habitats* (68%, 193 abstracts). The other three clusters grouped a third (32%) of the abstracts, with the cluster of *trade, use and conservation* having the lowest number of abstracts (8), followed by the clusters grouping research on the ecological effects of *climate and land-use change* (32) and *responses to disturbance* (52).

In the cluster of diversity across *human-modified habitats*, research focuses on evaluating the different aspects of diversity (species richness, abundance, species composition, biomass and others) across different anthropogenic habitats (e.g., secondary forests, plantations, urban areas, etc.) with a focus on specific taxa (e.g., orchids or bromeliads) but with diverse approaches. The cluster *responses to disturbance* concentrates on comparative research where differences at the community level are evaluated between old-growth forests and habitats that have undergone land-use changes of different types; this is similar to the cluster of ecological effects of *climate and land-use change,* with the difference that in most studies the ecological effects of land-use are evaluated but are mostly used as a proxy for possible effects of climatic changes. Finally, the cluster *trade, use and conservation*, is the most consistent, focusing on research on the use of species and the different aspects of their trade in the context of conservation.

The total number of words per cluster varied somewhat according to the number of abstracts (number of words for *trade, use and conservation*, 91; *climate and land-use change*, 352; *human-modified habitats*, 3575; *responses to disturbance*, 62; Supplementary Material, Figure S3), but this could also be influenced by the length of the abstracts per group, the stemming process whereby several words could be grouped under their stem (e.g., epiphyte, epiphytic, forest, forestry, etc.), or the fact that some abstracts contained a large number of deleted terms (e.g., prepositions, articles, adverbs, and conjunctions). The word abundance structures of clusters *trade, use and conservation* and *responses to disturbance* were more similar than they were to *climate and land-use change* and *human-modified habitats*, as they contained several common words (those with the highest frequency; for *trade, use and conservation* the three most frequent words were repeated between 210 and 353 times, and for *responses to disturbance* the three most frequent words were repeated from 419 to 1298 times, Supplementary Material, Figure S3 and Table S2), and these clusters had very few to no rare words (those with low frequency, e.g., repeated ≤ 20 times); while *climate and land-use change* and *human-modified habitats* had very many rare words (*climate and land-use change* with 43 singletons and 13 doubletons, and *human-modified habitats* with 1853 and 571, respectively, Supplementary Material, Table S2).

This means that clusters on *trade, use and conservation* and *responses to disturbance* are relatively consistent, independently of the number of publications/abstracts contained, or that the abstracts within these clusters somewhat consistently mainly reflect research on the impact of *trade, use and conservation* and *climate and land-use change*, respectively. The clusters on *human-modified habitats* and *climate and land-use change* grouped research abstracts with far fewer thematic or words in common. From these clusters, the one on *human-modified habitats* had the most heterogeneous content, and is almost miscellaneous, perhaps because the research into the anthropogenic effects on diversity can be very broad and is very heterogeneous for vascular epiphytes (e.g., lack of sampling protocols or general recommendations in the specific field). While the cluster on *climate and land-use change*, which mainly uses the ecological effects of land-use as a proxy to evaluate the effects of climatic changes on diversity, contains less explored avenues of research directly evaluating the impacts of climate change effects on vascular epiphyte diversity (one from 2002 and all others from 2009 till 2023), e.g., the modeling of forest dynamics, long-term dynamics, and experimental testing of the climate change effect (Supplementary Material, Figure S2, Table S1).

### 3.3. Tropical Forest Conversion and Epiphytes

Habitat loss caused by the growing human pressure on terrestrial ecosystems is one of the most important threats to biodiversity, especially in the tropics [9,70,71]. The planet is suffering rapid and dramatic changes across most biomes [72]. Considering the current high rates of deforestation in most tropical countries [73], it is projected that the few existing areas with extensive, undisturbed forests will become scarce and fragmented soon [5,9]. Human population growth and the intensification of agriculture are the major factors threatening primary forests in the tropics and their associated biodiversity [74]; due to conversion into cropland, grassland for cattle, and human settlements, many plant taxa have disappeared [75]. In spite of this bleak scenario, botanists should investigate poorly explored areas where species new to science can still be found, and possibly, species listed as extinct may be rediscovered [76].

**Figure 4:**
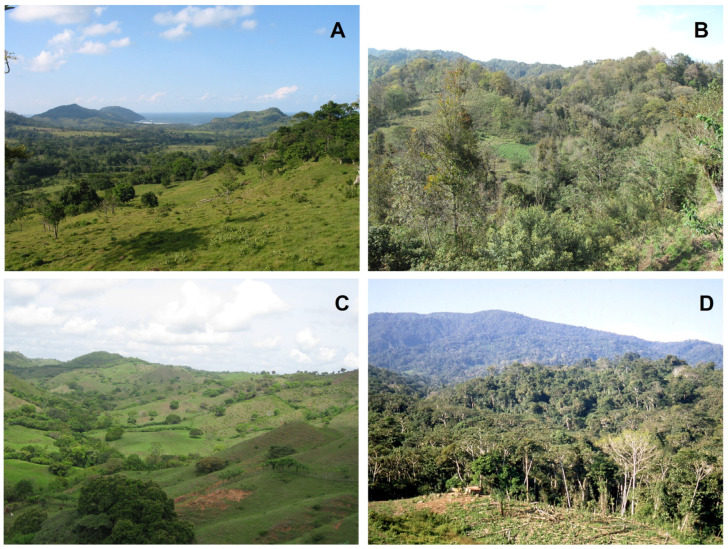
Views of human-modified landscapes in the Neotropics, including old-growth forest fragments, secondary forests, cattle pastures or plantations with remnant trees, and agricultural lands in (**A**) Los Tuxtlas, Mexico; (**B**) Central Veracruz, Mexico; (**C**) Azuero, Panama; (**D**) Alto Beni, Bolivia. Photographs by (**A**,**B**,**D**) Thorsten Krömer; (**C**) Helena J. R. Einzmann.

The conversion of primary or old-growth forests, i.e., forests that have never been clear-felled and have been impacted by little or no known recent human disturbance [5], in the tropics to treeless vegetation will result in an almost complete loss of epiphytes in the affected area [42], although an unknown number of facultative epiphyte species might survive on the ground or on human-made structures like walls, roofs or electricity wires [77,78,79]. In many human-modified landscapes (Figure 4), potential hosts may still be present, e.g., in the form of fragments of disturbed or secondary forests, isolated remnant or planted trees, or in plantations [46]. Although in the last two decades the number of studies considering the effects of various human activities on epiphyte diversity has increased considerably (Figure 2; Supplementary Material, Table S1), the fate of epiphytes in anthropogenic tropical habitats remains insufficiently understood. Therefore, a central question of tropical plant conservation remains whether human-dominated landscapes can provide suitable refuges for epiphytes, or whether these challenging habitat conditions result in slowly declining communities with strongly diminished regeneration, rendering them ‘living dead’ [80].

A few of these studies included three or more relevant types of anthropogenic habitats, comparing them with old-growth forests along gradients of human impact at a single study site, while considering all vascular epiphyte taxa in standardized study units in humid forests in the Neotropics [39,41,81]. We will take these comprehensive investigations as a starting point to explore the impact of land-use changes on epiphyte diversity in human-modified landscapes. It was observed that species richness was consistently the lowest in young secondary forests, followed by plantations or isolated remnant trees in pastures, disturbed forest fragments, and it was the highest in old-growth forest. We here conduct our review of the literature following this gradient in the decline of epiphyte diversity with increasing degrees of disturbance by starting with the most affected habitat type.

### 3.4. Secondary Forests

The conservation value of tropical secondary forests has been debated for decades [12,82,83]. Here, we define a secondary forest as a habitat that has recovered or regenerated largely through natural processes after significant human or natural disturbance (e.g., clearcut logging or hurricanes) that caused a complete removal of the original forest vegetation [82]. The remaining open area usually has been used as cattle pasture or for the cultivation of cash crops such as corn or sugar cane, but then due to various reasons (e.g., infertility of soils, low crop prices), these lands were not intensively used anymore, and as the result of secondary succession, a young fallow forest has started developing on the abandoned ground, which initially lacks resident epiphytes [82,84].

Considering trees or lianas, secondary forests may attain many aspects of the structure and species richness of old-growth forests within a few decades if there are propagule sources left at a feasible distance, but reaching a similar species composition may take centuries [15,85,86]. Some plant species, including many vascular epiphytes, however, appear to be old-growth specialists that only establish on the large host tree species present in old-growth forests [39,40,87]. This is likely because of the specific abiotic conditions needed (e.g., stable microclimate with high humidity and low insolation), which are only found in structurally complex forests [88,89].

**Figure 5:**
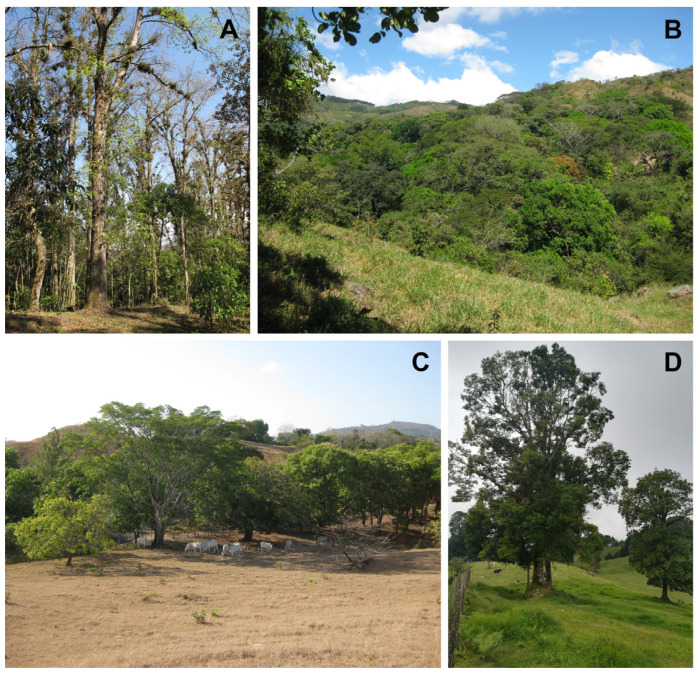
Views of two young secondary forests at (**A**) Central Veracruz, Mexico; (**B**) Veraguas, Panama; and cattle pastures with shade trees at (**C**) Azuero, Panama; (**D**) Central Veracruz, Mexico. Photographs by (**A**,**D**) Thorsten Krömer; (**B**,**C**) Helena J. R. Einzmann.

Generally, the destruction of old-growth forests results in a considerable species loss and a major decrease in total species richness, as is found in human-modified habitats [72,90], but this reduction may be more severe for vascular epiphytes than for other plant groups [37]. For example, in the few remaining patches of natural vegetation and mostly secondary rain forest fragments of Singapore, Turner et al. [19] found that epiphyte diversity was reduced by 62% compared to original flora, and was thus affected much more severely by local extinction (i.e., extirpation) than any other life form. This considerable reduction in the species richness of epiphytes following deforestation is corroborated by several studies, which indicate that fragments of young secondary forests (less than 20 years old; Figure 5A,B) usually show lower species richness of epiphytes in comparison with nearby sites of primary forests, while older secondary forests can recover a mayor part of that diversity [41,87,91].

**Figure 6:**
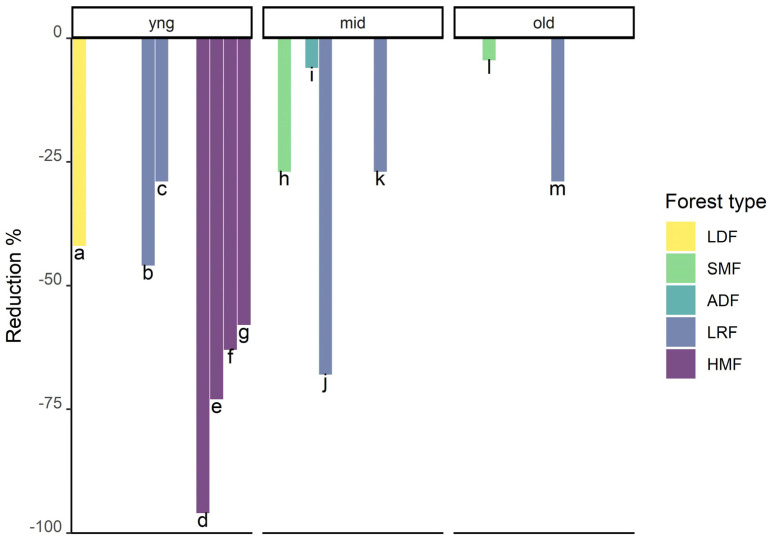
Epiphyte species-richness reduction in secondary forest fragments (young—yng < 25 years; middle—mid 25–50 years; old > 50 years) compared to old-growth forests (ADF—Andean dry forest; HMF—humid montane forest; LDF—lowland dry forest; SMF—subtropical montane forest; LRF—lowland rain forest) in different study regions in tropical countries: (a) Mexico [94]; (b, k) Colombia [91]; (c) Mexico [92]; (d) Venezuela [39]; (e) Bolivia [40]; (f) Ecuador [41]; (g) Mexico [81]; (h, l) Argentina [93]; (i) Ecuador [44]; (j, m) Panama [87].

For example, in three 15-year-old fallows, there was a reduction in species richness of between 61% and 73% in humid montane forests of Bolivia [40], and between 46% and 66% in comparable young secondary forests in Ecuador [41] and Mexico [81] and lowland rain forests of Colombia [91] (Figure 6; Supplementary Material, Table S3). In contrast, Barthlott et al. [39] found a 96% reduction in species-richness in a 23-year-old fallow in humid montane forests in Venezuela, whereas species numbers were less reduced in a similar aged secondary forest in lowland rain forests of Mexico [92], as well as in slightly older secondary forests in other humid forests in South America [41,91]. Finally, in the oldest secondary forests studied, epiphyte diversity was still reduced by 64% and 29% after 85 and 115 years of abandonment, respectively, in the humid lowlands of Panama [87], whereas in a 60-year-old subtropical montane forest in Argentina, epiphyte diversity had almost completely recovered [93].

Generally, the recolonization of secondary forest by epiphytes seems to be a slow process [46,87,95], but available data are hard to compare because of differences in the spatial context (e.g., distance of seed sources) [96,97,98]. Dispersal limitations may hamper the successful establishment of epiphytic bromeliads in human-modified habitats [99]. However, it is challenging to disentangle dispersal limitations from establishment limitations [100], although the clumped distribution of many epiphytes indicates that dispersal limitation might be involved [101].

The difference in both vascular epiphyte richness and species composition between young and old secondary forests is probably due to the unfavorable characteristics of young trees. On the one hand, they provide a more homogeneous structure with fewer microhabitats, a drier microclimate and lower bryophyte cover, and on the other hand, a shorter period for recolonization [81]. Differences in structural variables mainly lead to a loss of drought-sensitive hygrophilous species, with specific habitat requirements, such as shade- and humidity-adapted understory orchids and ferns, which might not be able to persist under modified microclimatic conditions (higher temperature and less moisture) in the open young fallow forests [102,103]. Similarly, *Socratea* palm trees were estimated to be c. 20 years old before colonization with vascular epiphytes began [104], while it took at least 10 years for epiphyte seedlings to recolonize stripped branches in humid montane forests [105]. In addition, most studied epiphytes show inherently slow growth; it may take more than 10 years for certain species to reach reproductive maturity [106,107].

However, two studies conducted in dry secondary forests indicated that the reduction in epiphyte richness, especially in young fallows, may be less severe (Supplementary Material, Table S3), as richness in 5–8 year-old and 10–20 year-old forests in the lowlands of Mexico was reduced by only 62% and 42%, respectively [94], and the epiphyte diversity of inter-Andean secondary dry forests of 13–28 years was almost comparable to that in a closed-canopy forests [44]. This relatively rapid recovery of epiphyte richness may be attributable to the physiological and morphological traits of the local drought-tolerant species that help them to cope with the harsh conditions in dry forests [108,109], whose canopies are usually low and open and provide little microclimatic buffering [110]. Many of the local species in these forests are widely distributed in Neotropical forests, because they are able to survive in a wide range of environmental conditions [93]. Moreover, dry forests in general have relatively poor epiphyte flora [21,44] that may recover after disturbance much faster than the highly diverse epiphyte flora with many rare species in humid montane forests [39,41,81].

Concerning the specific responses of major taxonomic groups, there are some trends, but substantial variance between studies warns against premature generalizations. For example, orchid richness in secondary forests of Bolivia decreased by ca. 90% compared to old-growth forests and by ca. 45% compared to ferns [40]. This study corroborates the findings from Singapore, where Turner et al. [19] found only 10 of the 110 inland epiphytic orchids (91% decrease), whereas the epiphytic pteridophytes were much less reduced in diversity (37%). In contrast, Barthlott et al. [39] documented a total loss of orchids and a 92% reduction in ferns in secondary forests in Ecuador, which had been completely cleared 23 years prior. Orchids were also the most affected epiphyte groups in two secondary forests in Mexico, showing a loss of >70% compared to adjacent old-growth forests [81,92]. However, the fate of ferns differed markedly; while the loss was about 65% in the humid montane forest, no reduction was found in the lowland rainforest. Also, Carvajal-Hernández et al. [111] found that in 10- and 20-year-old secondary forests in humid montane Mexico, epiphytic fern richness was reduced by 36% and 27%, respectively. The establishment of epiphytic orchids generally might be hampered by their dependence on mycorrhizal fungi for seed germination [112], as well as due to the unfavorable microclimatic and structural conditions in fallow forests [40]. The latter also affects many drought-vulnerable grammitid, vittarioid, and filmy ferns [39,89], while some species of the genera *Pleopeltis* and *Polypodium* have traits associated with drought resistance (e.g., poikilohydry, dense scales; [113,114,115]).

Similarly, studies on the human impact on bromeliads also showed mixed results. In humid montane areas, species richness was usually more reduced in young fallow forests (<20 years; 75–88% reduction) than in old-growth forests [39,40], while secondary forests of 30–40 years could recover some of the species richness present in old-growth forests (53–30% reduction; [116,117]). In contrast, in moist lowland areas in which the number of bromeliad species is generally lower, the species-richness of secondary forests was only slightly reduced compared to old-growth forests [91,92]. Moreover, secondary forests in dry lowlands can have even more species richness than old-growth forests [94,118].

Some bromeliad species, specifically atmospheric or xeromorphic species of the genus *Tillandsia*, can be more abundant in secondary than in adjacent old-growth forests [102,119]. These are well-adapted to anthropogenic habitats, which are more open and drier, through succulence, foliar trichomes, and CAM [114,120]. Apparently, the increased light levels in the canopies of secondary forests can favor the survival and growth of some less specialized, drought-tolerant *Tillandsia* species [121,122,123], which can dominate and increase their biomass, causing the epiphyte community to become less rich and less even [117,124,125].

### 3.5. Tree Plantations

Over the last several decades, commercial agriculture expansion has become a key driver of forest loss in the tropics [126]. Currently, agricultural production in tropical biomes has undergone a dramatic shift that has led to increases in the production of crops and wood products from plantations [127]. Agriculture plantations are defined as areas that are typically monocropped with perennials, producing tropical or subtropical products that commonly require prompt initial processing, and for which there is an export market [128]. Some of the most important tropical plantation crops are bananas, pineapple, sugar cane, and tobacco [129]. However, these agriculture plantations comprise herbaceous plants that can hardly host epiphytes, and typically are monocultures without remnant forest or cultivated shade trees.

Therefore, our discussion is focused on the impacts of different tree plantations on epiphyte species richness. Tree plantations can be defined as mono- or polycultures of agricultural arborescent species established and managed by humans for wood, fruit, luxury food, fiber and other products [130], e.g., oil and coconut palms, or coffee, cacao and tea under shade trees [131]. The expansion of tree plantation areas in the tropics has frequently come at the expense of intact forests, and increased from about 6.7 million ha in 1965 to 109 million ha in 2005 [132].

**Figure 7:**
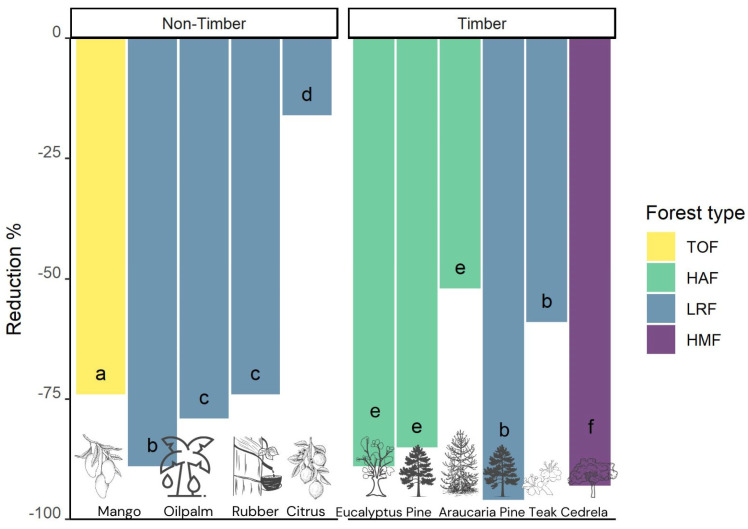
Epiphyte species richness reductions in different tree plantations compared to old-growth forests (HAF—humid Araucaria forest; HMF—humid montane forest; LRF—lowland rain forest; TOF—tropical oak forest) at different study regions in tropical countries: (a) Mexico [113]; (b) Panama [46]; (c) Indonesia [138]; (d) Mexico [92]; (e) Brazil [133]; (f) Venezuela [39].

Generally, these kinds of plantations are not known for their high capacity to maintain or harbor rich epiphyte communities, as studies mainly show reductions > 50% compared to nearby old-growth forest fragments (Figure 7; Supplementary Material, Table S4). First, we discuss structurally impoverished timber plantations with exotic pine or eucalypt species that usually host very few epiphytes [46,133]. Pines have been considered poor epiphyte hosts, not only because of the contents of phenolic and resinous substances [134,135,136], but also because of the instability and low water-holding capacity of their bark [137]. Additionally, the monopodial growth and lack of large horizontal branches of these conifers might be a constraining factor limiting epiphyte abundance and diversity [109].

Boelter et al. [133] compared epiphyte richness in a natural *Araucaria* forest of the Atlantic Forest in Brazil with that in managed plantations of *Araucaria*, *Pinus*, and *Eucalyptus*. The plantations of native *A. angustifolia* (Bertol.) Kuntze had a much higher conservation value than the exotic monocultures of *P. taeda* L. and *E. saligna* Sm. with reductions of 52% vs. 85% and 89%, respectively. While host trees of *A. angustifolia* are characterized by well-developed secondary branching that might favor epiphyte establishment and growth, the physiognomic and bark characteristics (e.g., crown morphology, textural, absorptive and physiochemical properties) of *Pinus* and *Eucalyptus* trees are mostly unsuitable [133]. In contrast, in an Andean 32-year-old plantation of the native *Cedrela montana* Moritz ex Turcz., epiphyte richness was reduced by >90% [39]. This was explained by the uniform tree structure, the absence of an understory and the poor development of plantation trees with a crown openness of more than 50%, which reduced the number of suitable micro-habitats for epiphytes.

**Figure 8:**
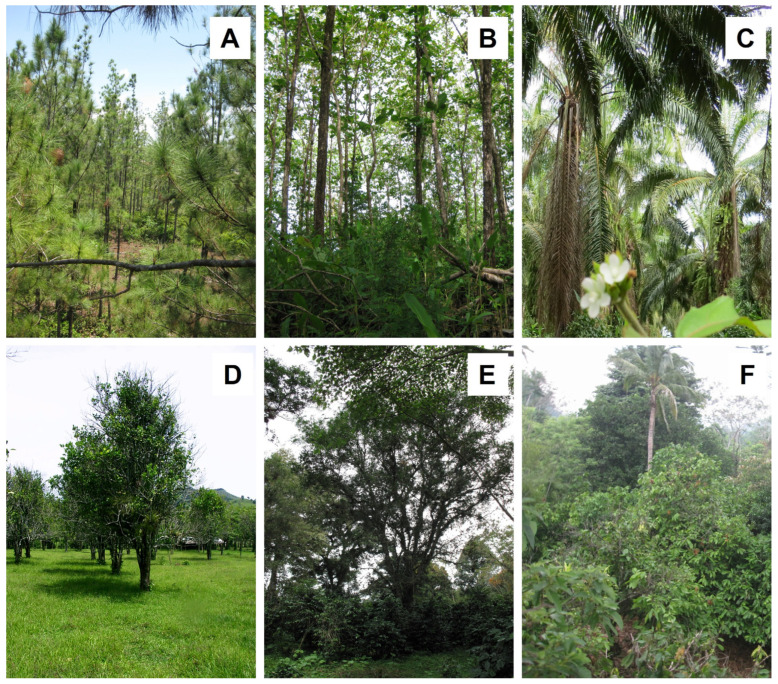
Views of different tree plantations of (**A**) pine in Veraguas, Panama; (**B**) teak in Veraguas, Panama; (**C**) oil palm in Chiriquí, Panama; (**D**) citrus in Los Tuxtlas, Mexico; (**E**) shaded coffee in central Veracruz, Mexico; (**F**) shaded cocoa in Tabasco, Mexico. Photographs by (**A**–**C**) Helena J. R. Einzmann; (**D**,**E**) Thorsten Krömer; (**F**) Jonas Morales-Linares.

In a comprehensive study, Einzmann and Zotz [46] found that pine (Figure 8A; *Pinus caribaea* Morelet) and teak (*Tectona grandis* L.f.; (Figure 8B) plantations hosted considerably fewer epiphytes than the tree stands surrounding them. The short rotation times of the timber crop leave little time for the colonization of these plantations. Consequently, the epiphyte individuals observed in the teak (and pine) plantations were mostly juveniles, and there were hardly any adult plants with reproducing structures [46].

The second types of tree plantations discussed are rubber (*Hevea brasiliensis* (Willd.) Müll. Arg.) and oil palm (*Elaeis guineensis* Jacq.; Figure 8C). The former produces latex sap, which is the basis for the natural rubber used mostly in the manufacture of automobile and aircraft tires, while palm oil, obtained from fruits and seeds, is mainly used in making cosmetics, biofuels, pharmaceuticals, and edible products. These are the two main crops threatening biodiversity and natural habitats in Southeast Asia and Latin America [139,140]; however, in the last decade, the decreasing prices and high labor costs in contrast to higher profitability are driving the conversion of rubber to oil palm plantations [141].

The first study considering the effect of land-use changes on epiphytes was published by Madison [69], who studied their distribution in a rubber plantation in Malaysia. When a rubber plantation is established, the forest is cleared and burned, and young trees from a nursery are planted. As the plantation matures, individual trees are colonized by epiphytes whose seeds arrive from populations in nearby primary forests or older plantations [69]. Of the 25 recorded epiphytes, orchids and ferns with minute diaspores dispersed by wind were the most species-rich and showed a random distribution in the plantation, similar to a few fleshy-fruited species dispersed by birds.

Almost four decades later, Böhnert et al. [138] studied the epiphyte richness in rubber and oil palm plantations in central Sumatra, Indonesia and found a reduction in epiphyte richness of 74% and 79%, respectively, compared with adjacent rainforest. They suggested three main factors as responsible, as follows: 1. a less favorable microclimate with hotter and drier conditions and more limited ecological niche space for a greater number of specialized epiphyte species; 2. the age and characteristics of the host trees, as these plantations are generally replanted after about 25–30 years, which abruptly stops the age-related increase in epiphyte species richness; 3. the occasional removal of epiphytes by plantation workers who commonly assume that epiphytes reduce yields by parasitizing the trees. Rubber trees are sparsely colonized by epiphytes anyway, and the removal drastically curtails any successful establishment of a new epiphyte community [138]. In contrast, the complex structure of oil palm stems hampers complete epiphyte removal, and larger individuals might be the most affected by this practice. In conclusion, the values of both monotypic tree plantations for epiphyte conservation are very low [138].

Apart from these studies, there is little and mostly anecdotal information on the suitability of oil palms as a host for epiphytes [142,143], although the accumulation of organic material in leaf bases makes them unique hosts compared to other plantation trees [144]. However, as epiphytes are mentioned as problematic in some management practice guides (e.g., [145]), they are regularly removed from oil palms [142], although Prescott et al. [144] showed that their removal did not improve the productivity of the crop and thus should be avoided, even from an economic point of view. Interestingly, oil palms are invasive in parts of Brazil, at least in part due to fruits that are attractive to many disperser animals, and have high germination success and establishment rates [146]. Notably, about a third of the epiphytes in a sub-spontaneous stand of *E. guineensis* were accidentals, terrestrial species that establish in the organic matter accumulated in their leaf sheaths.

Another case of cultivated palm trees is coconut (*Cocos nucifera* L.), which provides food, fiber, biofuels, cosmetics, medicine, and building material. However, coconut palms were shown to be poor epiphyte hosts, because of their smooth trunks, low structural diversity and usual occurrence in relatively dry, exposed contexts providing harsh microclimatic conditions; although a small number of ferns and at least one orchid species have been found on them [147,148].

The third type of tree plantation is cultivated only for the production of edible fruits, including important crops such as mango (*Magnifera indica* L.) and different species of *Citrus* (e.g., oranges, mandarins, lemons, grapefruits). Information on the suitability of mango trees for epiphytes is contradictory: Schimper [149] called them “poor” hosts in the West Indies, but “good” hosts around Rio de Janeiro. While the first categorization was supported in Johansson’s study in Africa [150], the latter was supported by observations in Costa Rica [151]. The study of Nir [151] provided evidence that large/old individuals hold disproportionately large epiphyte loads, while many smaller mango trees were entirely free of epiphytes. Hietz [42] found an epiphyte richness reduction of 74% in mango compared to nearby forest trees. One reason appears to be the very dense foliage concentrated in the outer crown, which is a strong barrier to propagules [149]. However, once colonization has occurred as a chance event, plants can thrive because humid conditions inside the crown are conducive to epiphyte growth, although little light might reach the inner branches [42,152].

There are also ambivalent results regarding the suitability of citrus trees for epiphytes. Hietz-Seifert et al. [43] noted that young (<25 years) *Citrus* spp. and *Cedrela odorata* L. trees cultivated together in a pasture were poor hosts. On a total of 45 individuals of both trees, only 15 epiphytic species could be found, equivalent to a reduction of 57% with respect to a nearby rainforest fragment in Mexico. In contrast, in the same region, Pérez-Peña and Krömer [92] recorded only a 14% reduction by recording 51 epiphyte species in up to 40-year-old *Citrus* plantations (Figure 8D) without epiphyte removal as a management practice, where mostly drought-resistant bromeliads and ferns had a higher richness than in the forest. Interestingly, the number of orchid species was also similar, which is usually the taxonomic group most negatively affected by disturbance and land-use change (e.g., [19,39,40,81]). However, it is known that citrus trees can be particularly good hosts for certain orchid species that can become very abundant on the smaller branches and twigs, such as *Ionopsis utricularioides* (Sw.) Lindl. [153,154].

**Figure 9:**
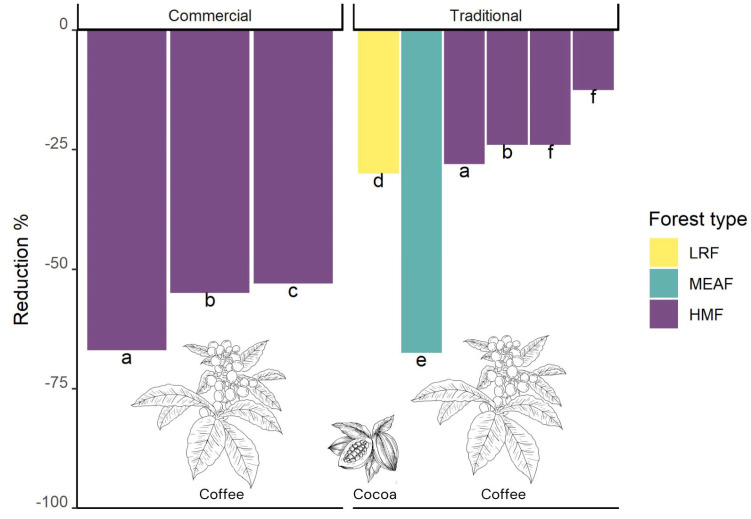
Epiphyte species richness reduction in different shaded coffee and cocoa plantations (Commercial monoculture with low density of shade trees; Traditional coffee polyculture with high density of shade trees) compared to old-growth forests (HMF—humid montane forest; LRF—lowland rain forest; MEAF—moist evergreen afromontane forest) at different study regions in tropical countries (a) Mexico [158]; (b) Mexico [162]; (c) Mexico [81]; (d) Ecuador [176]; (e) Ethiopia [164]; (f) Nicaragua [163].

The epiphyte assemblages in human-modified systems are typically qualitatively and quantitatively very different from those found in old-growth forest [20]; however, epiphyte diversity in plantations is not invariably low. Traditional coffee polycultures with shade trees in Latin America can have low reduction values compared to the other tree plantations described above (Figure 9; Supplementary Material, Table S4). The cultivation of coffee (different species of *Coffea*) can be considered as a specific type of tree plantation, as these consist of small shrubs or bushes that are periodically pruned to improve productivity and facilitate harvesting the ripened coffee cherries [155]. These are cultivated either in intensively managed sun plantations or in traditional agroforestry systems, grown under a more or less dense canopy of various natural humid montane forest or cultivated shade tree species [156,157].

While epiphytes almost never grow on coffee shrubs in sun coffee plantations [158], in shaded polycultures in southern Mexico, Mondragón et al. [155] found 23 species of vascular epiphytes, mostly polypodioid ferns and orchids; in Puerto Rico, Nir [151] found nine species of epiphytic orchids, and in a review on orchids in Mexican coffee plantations, Espejo-Serna et al. [159] reported 18 species that grew on the coffee shrubs themselves. In Ethiopia, the origin of coffee (*Coffea arabica* L.), Hylander and Nemomissa [160] found that large, ancient coffee bushes in home gardens carried a low diversity of vascular epiphytes similar to the surrounding old shade trees. Here, coffee occurs as a natural understory shrub within more or less intensively managed forest fragments [161]. Despite the generally low value of the coffee bushes themselves as hosts for epiphytes, plantations of shade-grown coffee carry great importance for epiphyte conservation because of the old shade trees that might host a similar diversity of epiphytes to trees in surrounding old-growth forests ([158]; Figure 8E). For instance, commercial coffee mono- or polycultures with small or a low density of shade trees showed a reduction of >50% compared to nearby forest fragments, whereas in traditional polycultures with many old shade trees, the reduction was only 12–28% [81,158,162,163]. However, epiphyte species richness can also be notably reduced (68%) on remnant shade trees in coffee agroecosystems as compared with forest trees in Afromontane forests of Ethiopia [164].

In general, epiphyte communities in coffee plantations are more homogeneous than in the forest, possibly because the agroecosystem structure is uniform (same tree species and size), with few long-lived trees and a drier microclimate, which makes them unsuitable for drought-vulnerable epiphytes [158]. Therefore, large shade trees and remnants of the original forest represent an essential refuge for some species of epiphytes that have adapted to the ecological and environmental conditions of the agroecosystem [158,165,166,167]. For example, shade coffee agroecosystems in Mexico harbored fewer species of hygrophilous orchids and ferns, which are more susceptible to disturbance, but more drought-tolerant bromeliad species, compared to humid montane forest habitats [81,167,168].

Unfortunately, the deliberate removal of epiphytes from shade trees in coffee plantations is a common management practice in Latin America [169]. This activity forms part of the maintenance of the shade trees to increase the availability of light for the coffee plants, and because epiphytes are considered as harmful parasites. Indeed, epiphyte removal from shade trees and coffee bushes might have a positive effect on coffee productivity as plants produce more flowers and fruits [169,170]. However, this practice leads to a depauperate agroecosystem, with negative impacts on the epiphyte community and its associated fauna [168,171,172].

Similarly, Hundera et al. [161] indicated a negative effect of forest fragmentation size and management on the diversity of epiphytic orchids in shade trees of semiforest coffee systems, as in large and small managed fragments, epiphyte richness was reduced by 30% and 55%, respectively, compared to old-growth forest. In these agroecosystems, the canopy layer is manipulated, shrubs are removed, and the herbaceous understory is cleared to reduce interspecific competition and increase coffee yield quality and quantity [161]. Even though some endangered orchid species may persist in small managed fragments, these cannot compete with the conservation benefit generated by extensive unmanaged Afromontane forests. Besides this, De Beenhouwer et al. [173] showed that coffee shrubs and their shade trees in managed forest fragments are a suitable habitat for only a limited set of orchid species. Thus, to conserve orchid diversity, it is necessary to avoid coffee management intensification in the remaining old-growth forest. At the same time, farmers should keep old canopy trees and tolerate epiphytes on their coffee shrubs.

Considering the numerous studies on shade coffee agroecosystems, there is minimal information regarding the diversity of epiphytes in shaded cocoa (*Theobroma cacao* L.; Figure 8F) plantations. These can generally be found where tropical rainforests have been lost or disturbed, and the current landscape is a mixture of secondary vegetation. In this pastureland for cattle grazing and agroforestry plantations, cacao grows under shade trees of native or cultivated exotic species [174]. While a study on non-vascular epiphyte diversity in Ecuador has shown that species richness was usually lower on cocoa trees than on natural tropical rainforest trees [175], there is no similar research for vascular epiphytes. In the same country, Haro-Carrión et al. [176] found that epiphyte richness on relict shade trees in rustic shade cacao plantations was reduced by 30% compared to large trees in adjacent forest fragments. Aroids, *Peperomia*, and ferns were less species-rich in plantations than in forests, while there were no differences in orchids and bromeliads, which shows that cacao agroforests may preserve a portion of epiphyte diversity but do not fully compensate for the loss of forest. Similar results were reported by Morales-Linares et al. [174] in Mexico, where the reduction in epiphytic orchids in cocoa plantations was 31% compared to tropical rainforest fragments. However, this study considered shade and cocoa trees, which both contributed to maintaining orchid diversity. Interestingly, more than 50% of these phorophyte species harbored ant gardens composed of up to 22 epiphytic species [177].

Similar to traditional coffee and cacao polycultures, ancient tea gardens in the Yunnan Province of Southwest China were established by the partial clearing of the original vegetation, where tea (*Camellia sinensis* (L.) Kuntze) has been planted under shade tree remnants of the natural tropical montane evergreen forests [178]. These agroforestry systems combine biodiversity conservation and natural resource utilization, and are essential habitats for many native plant species, including epiphytic orchids [178,179]. Wang et al. [180] recorded a total of 19 vascular epiphyte species, including 13 orchids, on a total of 343 top-pruned tea trees, while Wu et al. [181] identified 85 epiphytic orchid species (in 33 genera) on pruned tea trees, including several threatened species meeting the IUCN Red List criteria (in two 40 × 100 m^2^ plots each in three ancient tea gardens). The high richness of orchids on tea trees may be due to the preservation of a large number of shade trees, which can serve as a reservoir for propagules and possibly even host a greater variety of orchid species than the shorter tea trees [181]. In each case, ancient tea gardens were shown to maintain a high epiphytic orchid diversity, and to provide pollinator services for reproductive success and a suitable micro-environment for seed germination. Besides this, to date, only one study, conducted in northern Bengal, India, has investigated epiphyte diversity on shade trees of tea plantations [182]. The authors found a total of 6704 individuals belonging to 74 species of 20 families of vascular epiphytes, mostly holoepiphytes (62%), hemiepiphytes (20%), and a few accidentals. The study shows that epiphyte assemblages on shade trees of tea gardens have a high potential to contribute to epiphytic diversity in non-forest ecosystems of this region.

Although some plantations have great potential to host diverse epiphyte communities, this will almost always go along with the loss of species that are very vulnerable to changes in microclimate. As epiphytes are mainly forest species, the majority will cope better in plantations with a structure that resembles an old-growth forest with little intervention by humans. Unfortunately, this stands diametrically against the intention and practices mostly applied in this context. Timber production is often executed at a large scale and with relatively short rotation times, which will drastically reduce the potential for epiphyte assemblages to fully establish. Furthermore, fruit production involves an even more regular human intervention in the plantation, be it for harvest or maintenance, and epiphytes are usually seen as pests in this context, even further precluding their establishment.

### 3.6. Pastures with Remnant Trees

Much land in the tropics worldwide has been converted to pastures with remnant trees and planted isolated trees, interspersed with scattered forest patches (Figure 5C,D). These pasture trees are spared as a source of shade and fodder for cattle and other pasture animals [183], and incidentally act as islands for epiphytes in human-modified environments. Thus, individual trees are often described as the minimum habitat unit of epiphytes (e.g., [184]), which is certainly a valid concept in the case of isolated trees in pastures [20].

**Figure 10:**
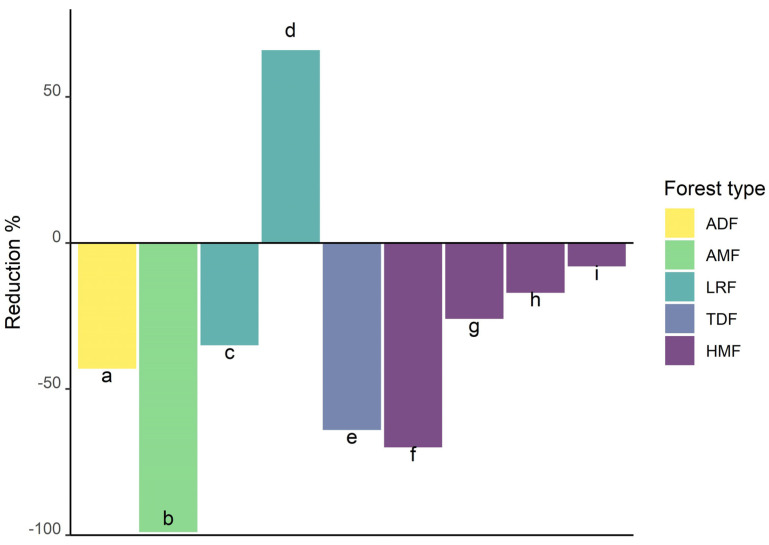
Epiphyte species richness reduction in isolated remnant trees in pastures compared to old-growth forests (ADF, Andean dry forest; AMF, Atlantic montane forest; HMF, humid montane forest; LRF, lowland rain forest; TDF, tropical dry forest) at different study regions in tropical countries: (a) Ecuador [44]; (b) Brazil [185]; (c) Panama [189]; (d) Mexico [43]; (e) Mexico [188]; (f) Ecuador [186]; (g) Mexico (2008) [192]; (h) Ecuador [41]; (i) Ecuador [45].

Studies of epiphyte communities on such isolated trees usually find a reduction in species richness compared to undisturbed forests (however, see [43]). However, this reduction is very heterogeneous (Figure 10; Supplementary Material, Table S5). By far the greatest loss reported, with almost no epiphytes found growing on isolated trees, comes from an area formerly covered by Atlantic Forest in Brazil [185]. It is likely that these isolated pasture trees were not remnants of natural forests, because the trees had significantly smaller diameters at breast height and smaller statures than trees in the studied forest. The long history of human modification of the Atlantic Forest region [185] might not only have led to the loss of the original forest, but also to the greater turnover of pasture trees, precluding the successful establishment of epiphyte communities in pasture trees.

Species loss at >50% was reported for epiphyte communities growing in pasture trees compared to their forest counterparts in montane forests of Mexico and Ecuador [186,187,188]. In contrast, Hietz-Seifert et al. [43] found more epiphyte species on pasture trees than on trees close to lowland rainforests in Veracruz, Mexico. The variability in vegetation zones, from dry to humid lowlands [43,189], and regions of lower humid montane forest [116,190], montane dry forest [44,191] and humid montane forest [41,45,192], could potentially explain the observed variation. However, even in the same vegetation zone, i.e., humid montane forest, in the same country, the recorded species loss varied by an order of magnitude between two studies, i.e., Larrea and Werner [45] vs. Werner et al. [186].

All studies, however, reported some extent of change in epiphyte composition, showing that assemblages on isolated remnant trees had a reduced floristic heterogeneity compared to old-growth forests, which is arguably an important aspect of land-use change in the context of epiphyte conservation [45]. Most studies concluded that a highly important driver is the change in microclimate towards higher levels of light and desiccation stress between the forest habitat and isolated trees that the epiphyte communities must cope with (e.g., [41,45,186,193]). An isolated tree cannot provide the same microclimate as a tree growing in a matrix of other trees; light air movement will diffuse humidity much more easily in an open pasture, and light will not only mainly penetrate the crown from above but also from all sides, thus reducing the diversity of habitat niches within the crown and changing the microclimate of the trunk considerably.

Hygrophilous species are usually the first species lost, such as filmy and grammitid ferns and other understory taxa (e.g., [40,192]), whereas more xerophytic taxa, adapted to drier climates (e.g., Bromeliaceae, Orchidaceae, Piperaceae, Cactaceae), usually cope well, and even increase in abundance, in such pasture habitats (e.g., [45,189]). Particularly drought-adapted bromeliads that thrive in the drier microclimates of isolated growing host trees are repeatedly observed making up a much higher percentage of the epiphyte communities than in trees from closed forests (e.g., [188,194,195]). This floristic turnover results in epiphyte communities with distinct species compositions compared to those of undisturbed forest habitats [45]. However, some communities appear to be dynamic, growing assemblages [194] that could be stepping-stones in time and space for re-populating nearby or newly forming secondary forests.

The diversity of the community of species on these hosts will depend highly on whether the trees are remnants of cut forests that already hosted epiphytes, or if the communities must establish from scratch. In both cases, the chance to increase diversity will depend on whether there are seed sources from which successful dispersal to the isolated trees is possible [99,105,117]. Also relevant is the constancy of the pasture trees. Much human intervention in the system will likely lead to the impoverishment of epiphyte communities, which could be the reason for the poor epiphyte diversity in pastures of the Atlantic Forest [185]. As short rotation times of tree plantations hamper the development of epiphyte communities, cutting isolated trees on pastures or in other human-modified landscapes will diminish their usefulness as stepping-stones for epiphyte communities, while closer seed sources will most likely increase the probability of re-establishment of epiphytes in any potentially growing secondary forest, as most epiphytes seem to have a high potential for long-distance dispersal [98]. Genetic evidence suggests that enough seeds of wind-dispersed epiphyte species enter the air column, allowing dispersal over several kilometers in fragmented landscapes [196]. On average, epiphytes have larger geographic range sizes than closely related terrestrial species, supporting the hypothesis that epiphytism favors dispersal into larger geographic areas. However, species in families where epiphytism is prevalent tend to have small range sizes regardless of their lifeform [197]. Nevertheless, dispersal in open landscapes may be much less limited than in forests [99]. In line with this notion, although dispersal limitation was repeatedly mentioned as a potential driver of their diminished diversity on isolated trees, it seemed to be a far less important driver than the change in microclimate (e.g., [41,45,186,193,198]).

### 3.7. Fragmented, Disturbed, Degraded and Managed Forests

The widespread deforestation in the tropics does not generally result in a straight frontier between old-growth forest and deforested land, but human activities encroach on the forest from many sides, which leads to a complex mosaic of old-growth forest fragments, negatively affected by edge effects, surrounded by secondary forests, agricultural land and deforested areas [42]. Understanding how the fragmentation and degradation of their natural habitats are affecting plants is critical for the conservation of biodiversity [199]. Thus, pertinent studies are urgently needed, as the conservation value of fragmented, selectively logged or otherwise degraded forest should not be taken for granted [45].

Forest fragmentation creates strong edge effects, abrupt ecological changes in previously continuous vegetation [200,201,202]. Haddad et al. [203] showed that approximately 20% of the world’s remaining forest area is within 100 m of an edge, while more than 70% of the planet’s forest areas are within 1 km from the next forest edge. The consequences of the edge effect include lower relative humidity, higher light intensity, and increased temperature and wind exposure [200,202,204]. Although the effect on the species richness of plant and animal communities has been extensively studied [205], only a few studies have focused on epiphytes [206,207,208,209], indicating reductions in species richness of up to 50% at the forest edge compared to inner forest sites in Brazil.

**Figure 11:**
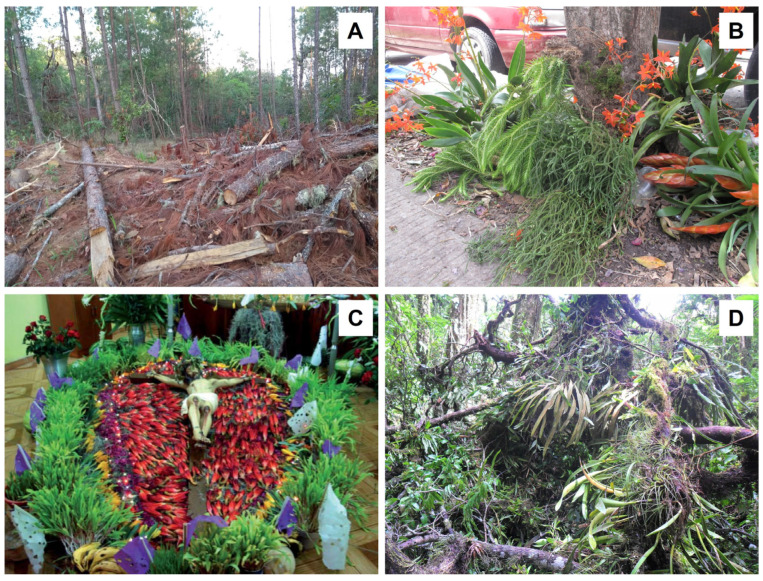
Views of (**A**) forest degradation by logging for timber in Chiapas, Mexico; (**B**) wild epiphyte traffic in the streets of central Veracruz, Mexico; (**C**) ceremonial use of epiphytes in Oaxaca, Mexico; (**D**) fallen epiphytes in Los Tuxtlas, Mexico. Photographs by (**A**) Nayely Martínez-Meléndez; (**B**) Thorsten Krömer; (**C**) Gabriela Cruz Lustre; (**D**) Esteban Francisco-Ventura.

In a similar way, forest degradation occurs as a result of human activities; it can be defined as a state of anthropogenically induced arrested succession, where ecological processes that underlie forest dynamics are diminished or severely constrained [210]. The degradation process can be rapid, or may take place over a long period and only become evident gradually [211], but it finally leads to a reduction in biodiversity and changes to the structure and species composition of the forest [212]. It is estimated that more than 2 billion ha of forests are degraded globally [213], while from a remaining area of 1071 million hectares of tropical moist forests, about 10% were degraded in 2019 [214]. The primary causes of forest degradation in the tropics are unsustainable exploitation, such as harvesting for timber, firewood and charcoal (Figure 11A), uncontrolled forest fires, and grazing [215]. Furthermore, the overharvesting of non-timber forest products (NTFPs), i.e., wild plants that are used for food, medicine, construction materials, and fibers, can have negative effects on the plant communities and lead to the disturbance of tropical forests [216]. Using the classification of Guzmán-Jacob et al. [109], disturbed or degraded forests are defined as fragments with clear signs of previous logging, sometimes with ongoing cattle grazing, the removal of understory and/or the harvesting of NTFPs. In contrast to secondary forests, these have not been clear-cut, and thus maintain at least some of the original canopy trees, but the structure, processes, functions and dynamics in degraded forests have been altered [212]. One of the main drivers of the degradation process in a forest is the selective harvesting of economically important trees [212]. Consequently, there is a need to adopt sustainable forest management practices that promote biodiversity conservation together with the production of timber [217,218].

Forestry management consists of programmed harvesting to achieve continual timber yield while maintaining considerable forestry carbon stock. In this way, a significant proportion of the forest’s structural attributes are preserved, allowing for the provision of those ecosystem services unmanaged forests provide [218]. Nevertheless, different methods of forest management, such as selective harvesting and rotation with intermediate felling, modify the composition and structure of tree vegetation [218,219,220].

**Figure 12:**
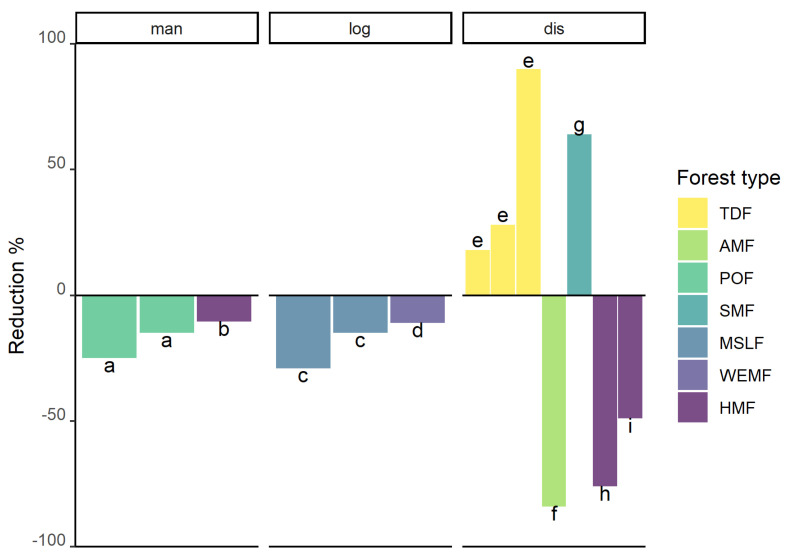
Epiphyte species-richness reduction in managed (man), selectively logged (log) or disturbed (dis) forests compared to old-growth forests (AMF—Atlantic montane forest; HMF—humid montane forest; MSLF—moist, semi-evergreen lowland forest; POF—pine–oak forest; SMF—*Sabal mexicana* forest; TDF—tropical dry forest; WEMF—wet-evergreen montane forests) in different study regions in tropical countries: (a) Mexico [218]; (b) Ecuador [45]; (c) India [241] (d) India [240]; (e) Mexico [119]; (f) Brazil [185]; (g) Mexico [242]; (h) Venezuela [39]; (i) Mexico [81].

The impact of forest disturbance in the context of different silvicultural management practices on the diversity of vascular epiphytes has been investigated in several studies with mixed results (Figure 12; Supplementary Material, Table S6). For disturbed humid montane forests, severe species richness reductions of about 50% and higher were reported. In the Atlantic Forest of Brazil, Parra-Sanchez and Banks-Leite [185] found that besides the drastically reduced number of epiphytic species (−84%), human-modified forests had low numbers of seedlings and juveniles, which will exacerbate the differences compared with continuous old-growth forest in the future, because of the high seedling mortality and lower tolerance to drought of early ontogenetic stages.

In the Venezuelan Andes, Barthlott et al. [39] found a sharp difference in species richness between a primary and a disturbed forest, although the last selective logging occurred some 50 years ago. The decrease in species richness of 76% at the disturbed site was driven mainly by the loss of epiphytes that grow in large stands and mats, which build up reservoirs of nutrients and water exploitable by many other epiphytes [39]. Consequently, epiphytes in the disturbed forest mostly grew on the bare bark.

Another severe reduction in epiphytic species (49%), mainly orchids, was found by Krömer et al. [81] in a disturbed humid montane forest in eastern Mexico, located in close vicinity to a large urban area, which had been used for the extraction of firewood, construction timber and NTFPs for decades. Specifically, the mostly illegal harvest of orchids for commercial and cultural purposes further reduced epiphyte abundance.

Due to their horticultural and ceremonial value, wild epiphytes are often sold in markets and streets in Mexico ([221,222,223,224,225]; Figure 11B,C), and in many other tropical countries [226]. In Africa and Asia, many epiphytic orchids are used in traditional medicine or as food supplements [227,228,229,230]; in addition, wild-collected orchids are commercially traded globally [231]. However, only a few studies evaluated the ecological effects of harvesting epiphytes [232,233,234,235]. Most of them show that sustainable use is difficult [236]. Other studies recommended taking advantage of the naturally fallen epiphytes in forests and agroecosystems ([169,233,237,238]; Figure 11D).

In another study from southern Mexico, Wolf [135] compared epiphyte diversity between sites of least disturbed old-growth pine-oak forests with cyclically clear-cut and selectively logged forests. Epiphyte richness in the ca. 25-year-old oak coppices with regular clear-cutting was 51% lower than in old-growth forests. Thus, cyclical clear-cutting should be avoided, as the restorative period is apparently too short for epiphytes to fully re-colonize coppices. In contrast, selectively logged pine-oak forests, in which large trees were spared, sustain epiphytic vegetation very similar in richness and biomass to old-growth forests [135]. Such remnant trees are essential for epiphytes that require the presence of accumulated suspended soil, and they may also serve as epiphyte seed sources for the re-growing trees in the vicinity. The author concludes that selective logging may help to protect the epiphyte vegetation locally, but if such management is not adopted over a larger region, much of the epiphyte species diversity in commercially exploited pine-oak forests will be lost.

In the same area, Martínez-Meléndez et al. [218] studied the epiphyte diversity in two pine-oak forest stands that had been subjected to thinning and release cutting (methods of silvicultural management) in comparison to an old secondary forest, with no timber harvest since the 1960s. While thinning only slightly reduced species richness by 15%, release cutting led to a 25% reduction, compared to the secondary forest. The latter is related to the lack of mature oak trees, which provide more favorable structural conditions (e.g., a variety of bark types) for successful epiphyte establishment [218]. In contrast, the predominating pine trees (e.g., *Pinus oocarpa* Schiede ex Schltdl., *P. maximinoi* H.E. Moore) are considered less suitable host trees for most epiphytes [134,135,137], although Jiménez-Bautista et al. [239] found some orchids established on *P. ayacahuite* C. Ehrenb. ex Schltdl.

In humid montane forest in northeastern Ecuador, Larrea and Werner [45] found that epiphyte species richness was only slightly reduced by 10% in managed forest with altered under- and midstory due to cattle grazing relative to old-growth forest without major human interference for at least 50 years. However, the managed habitat differed considerably in floristic composition from the old-growth forest, suggesting rapid species turnover in the course of only six years since cattle disturbance started, as shown by the loss of numerous drought-sensitive species, e.g., the replacement of ferns by more xerotolerant taxa [45]. It is likely that drought-sensitive species, as a functional group, hold a disproportionate share of rare and range-restricted species, thus the conservation value of intensively managed habitats should be viewed with caution.

Similarly, Seshadri et al. [240] showed a reduction of 11% in species richness between selectively logged (40 years ago) and unlogged wet-evergreen montane forests in the southern Western Ghats, India. Although there was higher epiphytic diversity and relative abundance in the selectively logged forest, the vascular epiphyte assemblage did not match that of the unlogged forest, e.g., there was a greater abundance of drought-resistant species. This goes in line with the study of Padmawathe et al. [241] in a moist lowland forest of the Eastern Himalaya, India, where they found in selectively logged forests a slight reduction of 15% in epiphyte richness and abundance, except for orchids, whose survival was ensured by large remnant trees. However, logging reduced the forest structural complexity and altered their microclimate, which negatively affected the abundance and species composition of pteridophytes and non-orchid angiosperm epiphytes, whose diversity can be maintained only if patches of forests are left uncut in logged areas.

In contrast, Flores-Argüelles et al. [119] found that in the three types of tropical deciduous and semideciduous lowland dry forests in western Mexico, surprisingly, selectively logged and grazed sites presented a greater number (18%, 28% and 90% increase) and abundance of epiphyte species than the adjacent old-growth forest sites. This could indicate that disturbances rather diversified the microhabitat types available in these dry forests, providing the possibility for other species types to establish (i.e., other than drought-tolerant species; [108,109]). A similar observation, i.e., slightly higher epiphyte richness in degraded vs. adjacent old-growth forest, was also made by Guzmán-Jacob et al. [109] at a 500 m elevation at the Cofre de Perote in central Veracruz, Mexico. Furthermore, Aguirre et al. [242] recorded a greater richness of epiphytes (64% increase) in disturbed sites compared with conserved sites in a forest dominated by the palm *Sabal mexicana*, on the Gulf Coast in Veracruz State. This pattern was mainly due to the accumulation of hemiepiphytic species of low abundance (e.g., *Ficus* spp.) and the presence of accidental epiphytes, whose growth was favored by the rich and abundant aerial substrate retained in old leaf bases of these palms located in open areas.

The impacts of forest fragmentation due to land-use change and degradation on vascular epiphyte species richness and composition boil down to significant reductions in epiphyte richness when compared to possible old-growth forests nearby, and shifts in species composition. Generally, the degree to which epiphytes are negatively affected depends on the severity of disturbance and the age or size of the remnant trees [125]. In addition to total species numbers, disturbance affects species composition, as anthropogenic disturbances force a shift from hygrophilous or mesic to more drought-tolerant species [45], but also vertical distribution, as epiphyte species from shady canopy strata decline in more open vegetation types compared with old-growth forests [43,158]. Activities such as selective logging, clear-cutting, cattle grazing, and the overharvesting of non-timber forest products (NTFPs) are actions that are largely carried out in already impacted ecosystems, or may be the direct reason for their conversion. Indirectly or actively, these activities create edge effects, and alter the structure and microclimate of forests. Because recovering ecosystems or secondary forests are here to stay, and because they have a high conservation value for terrestrial vegetation, it is still important to evaluate what possible changes in selective logging practices may be put into place to avoid the further degradation of epiphyte assemblages, along with research that can really establish what is the actual conservation value of remnant trees, as they are implied to be critical for preserving epiphyte diversity and maintaining ecosystem functions (although there is no evidence of the latter).

Generally, the degree to which epiphytes are negatively affected depends on the severity of disturbance and the age or size of the remnant trees [125]. In addition to total species numbers, disturbance affects species composition, as anthropogenic disturbances force a shift from hygrophilous or mesic to more drought-tolerant species [45], but also their vertical distribution, as epiphyte species from shady canopy strata decline in more open vegetation types compared with old-growth forests [43,158]. For epiphyte conservation purposes, it is recommended to adopt a management of selective logging instead of periodic clear-cutting, and to spare large trees during logging [135]. These remnant trees may be a “refuge” where a substantial portion of local floristic richness can survive and later serve as propagules, which otherwise would be lost during logging activity if not properly managed [243]. However, it is recommended that fallen epiphytes be rescued from the logged trees or the ground for possible translocation and reintroduction into nearby forest fragments as an enrichment strategy within ecological restoration projects [244,245,246,247,248].

### 3.8. Epiphytes in Urban Settings

Human settlements and infrastructure account for an ever-increasing, albeit still relatively small, portion of the global land surface (c. 0.2 × 10^9^ ha or c. 3% of the total), although the boundary between rural and urban areas is often blurred. Recent decades have seen numerous studies on the plant communities that appear spontaneously in these urban habitats [249]. Vascular epiphytes have not escaped the attention of scientists interested in the relatively new discipline of urban ecology, and over the last two decades, epiphytes in urban settings have been the focus of a considerable number of studies.

**Figure 13:**
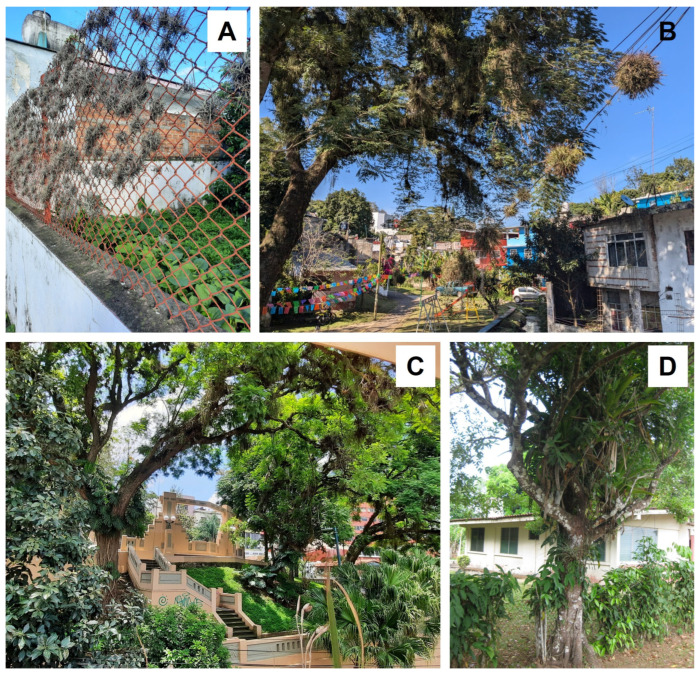
Views of (**A**) *Tillandsia schiedeana* Steud. on a mesh wire fence in central Veracruz, Mexico. (**B**) Epiphytes on power cables in central Veracruz, Mexico. (**C**) Epiphytes on park trees in central Veracruz, Mexico. (**D**) Epiphytes on fence tree in Las Lajas, Panama. Photographs by (**A**,**C**,**D**) Helena J. R. Einzmann; (**B**) Thorsten Krömer.

These studies cover numerous types of urban habitats (Figure 13), from urban forest fragments [250] to parks [251], college campuses [252,253], botanical gardens [254], private gardens [255], and single roadside trees [256,257], or deal with epiphytes on artificial substrates like electricity wires [79] or on buildings [78]. Although most of these studies were performed in America (e.g., [252,258,259,260]), there are reports from all over the globe, e.g., from Africa [261], Europe [262], Asia [257], Australia [263] and New Zealand [264]. The studies provide important baseline information, but a common disadvantage of most of these studies is their entirely descriptive nature, along with a lack of context—in most cases, there is no comparison of the urban epiphytic vegetation with that of an old-growth forest in the vicinity standardized for area or number of trees. Comparisons are also hampered by the frequent taxonomic bias, e.g., on orchids [253,265], bromeliads [266,267] or ferns [268,269].

A few studies, however (e.g., [152,257]), applied a comparative approach. Adhikari et al. [257] compared the species numbers and abundance of epiphytic orchids along a gradient of naturalness from single trees in urban settings to trees in nature reserves along Kathmandu Valley—urban settings’ median richness was 5% that of the nature reserves, while median abundance was also much lower (with c. 30%). Einzmann et al. [152] used a similar approach in southwestern rural Panama, and compared epiphyte diversity and abundance on trees in human settlements with those on trees in pastures and forests. Species numbers derived from rarefaction curves suggest a two-fold difference between the species numbers of urban habitat and nearby forest, but neither the average number of species per tree nor their abundance were significantly different. Aoki-Gonçalves et al. [266] analyzed epiphyte diversity and abundance in the city of Xalapa along a woody cover gradient from 10 to 100% in a 100 m buffer. As expected, epiphyte species richness was positively related to woody cover, but surprisingly, community composition was not. The most abundant species was *Tillandsia recurvata* (L.) L., a generalist species that is very common in urban contexts in much of the Americas. A study by Alvim et al. [270], which compared epiphytes in 26 urban green areas in Brazil, is highly instructive in this context. They reported a set of dominant species like *T. recurvata* or *Pleopeltis pleopeltifolia* (Raddi) Alston, with low diversity and a large proportion of accidental epiphytes, suggesting that the urban environment acts as a strong filter for widely distributed species, thus resulting in the homogenization of the potential regional epiphyte flora.

Most studies report the results of single censuses without information on size structure, which does not allow us to predict the longer-term perspectives of epiphytes in urban habitats. Again, there are exceptions; Mondragón and Mora-Flores [271] analyzed the size class structure of two *Tillandsia* species in the city of Oaxaca, Mexico. At least for *T. recurvata*, the large proportion of seedlings and juveniles suggests a dynamic population [272]. Clearly, repeated censuses are needed to obtain information on the dynamics of urban epiphytes. Results will certainly differ among species. A genetic study with *T. recurvata* highlighted the different consequences of the low connectivity of urban populations for selfing and outcrossing species [267].

Among the tree flora of many cities, exotic species are not uncommon. There is no indication that these differ from native trees as regards suitability as potential hosts (e.g., [253,271,273]). Quite a few species even seem to be particularly good hosts. For example, *Samanea saman* (Jacq.) Merr. from South America, which is frequently planted along roads in the city of Singapore, has a very rich epiphyte flora [274]. Similarly, locally exotic palm trees of the genera *Phoenix* or *Elaeis* were the focus of several studies because of their conspicuously rich epiphyte flora [275,276,277]. Noteworthy, a large proportion of the recorded epiphytes were actually accidentals. This observation is not idiosyncratic for palms—numerous studies of epiphytes in urban settings report a large proportion of accidental epiphytes (Table 1). Further increasing the heterogeneity of the epiphyte communities in urban settings is the occurrence of exotics, e.g., *Dendrobium nobile* Lindl. or *Platycerium* spp. in the Americas, where they have escaped from cultivation, or ornamentals may have intentionally been attached to trees [274,278,279,280].

In summary, the currently available evidence leads to the unsurprising conclusion that urban areas are quite impoverished in epiphyte diversity and abundance compared to a forest of similar size and location. Moreover, accidentals play a much larger role than in undisturbed vegetation. Nevertheless, there is a substantial number of epiphyte species, which led Neo et al. [281] to call cities “a botanical oasis rather than a biological desert”. We partially agree that, far from being diverse or hotspots, urban areas can play a certain role in epiphyte conservation.

**Table 1:** Epiphyte communities in urban settings. Given are the total numbers (#) of species growing epiphytically, the total and relative numbers of accidental epiphytes and exotics, the study site and the source.

Total # Species	Accidental #	Accidental %	Exotics	Study Site	Source
10	1	10	-	Piratininga, Brazil	[256]
8	2	25	1	Port Harcourt, Nigeria	[260]
16	4	25	-	Bogor, Indonesia	[282]
49	18	36.7	8	Mar de Espanha, Brazil	[283]
43	16	37.2	-	Juiz de Fora, Brazil	[252]
110	46	41.8	30	Juiz de Fora, Brazil	[270]
47	21	44.7	-	Juiz de Fora, Brazil	[258]
15	9	60	-	Buenos Aires, Argentina	[284]
72	48	66.7	-	Douala, Cameroon	[276]
34	23	67.6	12	Santo Domingo, R Dominicana	[285]
71	60	84.5	32	Quito, Ibarra, Riobamba, Mendoza; South America	[275]

## 4. Conclusions

This review provides a comprehensive overview of the literature on the impact of land-use change on vascular epiphytes, and thus spans individual studies that often focus on a locally and temporally restricted scale. Most studies across forest types agree that the conversion of tropical old-growth forests, typically into a mosaic of secondary forests, cattle pastures with remnant trees, and plantations of native exotic tree species leads to a general decline in epiphyte richness (e.g., [39,41,46,81]). However, the magnitude of the impact depends on the magnitude of disturbance and the type of vegetation that replaces the original forest, and varies with functional types. The associated changes in microclimate are much more severe for hygrophilous species in constantly humid ecosystems, such as lowland rain or montane cloud forests, while more drought-resistant species in less rich dry forests seem to be less affected, or may even benefit from these changes [44]. Three large groups, bromeliads, ferns, and orchids, seem to show distinct patterns regarding human disturbance when analyzed separately [40,45]. This is not very surprising; epiphytes are simply defined by their structural dependence on trees, but are functionally highly diverse [286]. Thus, we cannot expect a consistent response across all species.

The impact of climatic changes on vascular epiphytes, though still a relatively underexplored field, is increasingly being studied experimentally. The existing small body of literature indicates that epiphytes are affected by shifts in climatic conditions, particularly changes in precipitation patterns and the frequency of extreme weather events [287,288]. This will influence epiphyte diversity and community composition, with observations suggesting that canopy-dwelling plants are often, but not always, more vulnerable to such changes than terrestrial counterparts [35]. Although few studies have directly addressed the effects of climate change on epiphytes, evidence from various sources supports the notion that sustained microclimatic changes, regardless of disturbance intensity, will adversely affect many epiphyte populations [21,48,289]. Considering this, disturbances, which have long been used as a proxy for understanding potential climate change effects, are examined as factors contributing to the vulnerability of epiphytes to both natural and human-induced environmental shifts [290]. This review highlights how land-use changes—such as deforestation and conversion to agricultural land—impact epiphyte diversity across a wide range of systems. We assume the mechanisms in many cases are related to the drastic alteration of the microclimate, making the habitat unsuitable for epiphytes while allowing terrestrial vegetation to persist. This is because, unlike terrestrial plants, epiphytes are more dependent on their host trees and specific microclimatic conditions [95]. Hence, the regeneration of vascular epiphytes is slower than that of terrestrial plants, and this difference in recovery rates is essential when considering the ecological consequences of land-use changes for epiphyte populations.

While the value of secondary forests for biodiversity is increasingly acknowledged, none of these ecosystems entirely replicate the biodiversity of undisturbed primary forests. Nevertheless, they provide critical habitats for metapopulation persistence, but also contribute to carbon sequestration. Over time, secondary forests may assist in the recovery of a substantial portion of species richness, taxonomic diversity, and functional roles, including threatened and endemic species. Additionally, they complement primary forest remnants by enhancing landscape connectivity and offering alternative habitats. Even so, land-use change in tropical forests typically leads to substantial biodiversity loss, reduced ecosystem services, and decreased soil health. Primary forests are irreplaceable for biodiversity conservation, and efforts to mitigate land-use impacts should focus on preserving these areas and implementing sustainable land-management practices [291,292,293]. This irreplaceability paints a bleak scenario for plant biodiversity in tropical forests. The scenario is even worse for vascular epiphytes, which face several challenges regarding reestablishment after disturbance related to low dispersal, drought sensitivity, high mortality, and unsuitable substrates, microsites, or habitats. Also, epiphytes need not only terrestrial vegetation, but also a degree of habitat diversity in the forest so as to find suitable habitats, colonize, and recover once the challenges they face are overcome.

## 5. Future Directions

The plethora of methods and sampling designs applied when evaluating the effects of land-use on epiphyte diversity prevents a quantitative analysis of the different results [294,295]. To allow such analyses in the future, ecological studies on epiphytes require standardization; also, greater replication within and between trees, sites, and habitats is needed for a more rigorous statistical and quantitative assessment of the impact. Researchers should take measurements of climatic variables at sampling locations both within and outside of tree crowns, along the vertical gradient, with attention paid to the edge effects on epiphyte communities. Much more long-term research is needed to understand the consequences of disturbance on epiphytes at larger spatial scales and longer timescales [68]. The results of the few published long-term studies (e.g., [194,296,297]) suggest that epiphyte communities at a plot scale of, e.g., 1 ha are not at equilibrium. Thus, changes in secondary habitats may be unrelated to disturbance, but simply represent typical dynamics of epiphyte communities. An interpretation of such studies is only possible with similar studies in undisturbed forests.

Degraded habitats are becoming increasingly prevalent in tropical ecosystems. Therefore, it is key to understand the baseline of vascular epiphyte assemblages in natural habitats and across degraded ecosystems, as well as what temporal changes occur in degraded or non-natural ecosystems, so as to best inform restoration efforts. Although it is difficult to draw an overall picture of the impact and, therefore, solutions, some studies on the ecological restoration of degraded and secondary forests with naturally fallen epiphytes or those collected from felled trees reported encouraging results [244,245,246,247]. Active interventions or restorations seem to be more effective than a passive approach. For instance, active interventions are mentioned for areas with slow natural regeneration due to long disturbance histories, whereby actively planting vegetation nuclei may produce seed sources, accelerate recovery, and establish vegetation nuclei to mitigate dispersal limitation. Any effort should recognize and acknowledge the value of vascular epiphytes as secondary foundation species, whereby any active restoration would consider the species’ ecotypes and requirements.

For successful restoration with or without transplants, it is essential to understand abiotic and biotic influences (e.g., rainfall, drought, substrate conditions, branch and bark quality) on survival, colonization, and extinction. Vegetation restoration may enable the restoration of canopy vegetation. For instance, the transplantation of vascular epiphytes to host trees can be effective, with high survival rates influenced by species traits and environmental conditions. Focusing on particular “ecotypes” according to the forest’s stage of recovery could be a strategy, whereby tank bromeliad transplantation offers cost-effective support by creating stable microhabitats. Case studies, such as in the Colombian Andes [247], highlight the importance of host tree selection and considering epiphyte species-specific responses when transplanting.

None of these measures guarantee success without knowing the spatio-temporal dynamics. Temporal dynamics are almost unexplored, whether in old-growth forests or in degraded or secondary forests. We know very little about the long-term temporal changes of vascular epiphyte communities; therefore, long-term monitoring to evaluate the temporal variation of epiphyte assemblages at a fine-grain and broad-grained level is key to understanding how to aid regeneration and restoration best. Epiphyte survival and demographics show substantial variation over time, driven by both abiotic and biotic factors.

As a final thought, although our review focuses on vascular epiphytes, it seems essential that the research on the impact of land-use on vascular epiphytes be expanded to understand the cascading effects that the loss of vascular epiphytes may have on other components of the ecosystem.

## Data Availability

In accordance with Open Science communication practices, the authors declare that all data are available within the manuscript and Supplementary Materials.

## Supplementary Materials

The following supporting information can be downloaded at: https://www.mdpi.com/article/10.3390/plants14081188/s1, Figure S1: Visual assessment of the different methods for determining the optimal number of clusters for k-means; we used two types: total within sum of square or “wws” (upper graph) and gap statistics or “gap_stat” (lower graph) as a function of clusters. These methods include a direct method, consisting of optimizing a criterion, such as within-cluster sums of squares, and statistical testing, consisting of comparing evidence against null hypothesis, respectively; Figure S2: Four aspects of research: A, *Trade, use and conservation*; B, *Climate and land-use change*; C, *Human-modified habitats*; D, *Responses to disturbance*. Here we show the 20 most frequent words per cluster derived from the overall word matrix (based on all abstracts), independently of their frequency or the number of papers represented in each cluster. Words varied in their frequency per cluster; these are depicted in the Supplementary Material, Figure S3; Figure S3: Word clouds of the four aspects of research (upper graph): *Trade, use and conservation* (upper left, word input from eight references, see Table S1), *Climate and land-use change* (upper right, word input from 33 references), *Human-modified habitats* (lower left, word input from 195 references; an inset (lower graph) is provided for this category for the sake of visibility), *Responses to disturbance* (lower right, word input from 52 references). Word size reflects word frequency in relation to other words of the respective word cloud; Table S1: List of references resulting in four clusters of the following aspects of research: A, *Trade, use and conservation*; B, *Climate and land-use change*; C, *Human-modified habitats*; D, *Responses to disturbance*; Table S2: Comparison of the frequency of words in the different clusters (A, B, C, D), the frequency indicates the number of times the word is repeated in a given cluster, and it ranges from 1 (singletons) to 1298, whereby some cluster have a large number of common words (A and D) and others a large number of “rare” words or singletons (B and C); the differences in the abundance structure of the words per cluster can be contrasted graphically with Figure S3; Table S3: Comparison of epiphyte species richness reduction in old-growth vs. secondary forest fragments (SFF) at different study regions, indicating vegetation type (VT), elevational range (ER), annual precipitation (AP), and references in chronological order; Table S4: Comparison of epiphyte species richness reduction in old-growth forest vs. different types of plantations at different study regions, indicating vegetation type (VT), elevational range (ER), annual precipitation (AP), and references in chronological order; Table S5: Comparison of epiphyte species-richness reduction in old-growth forest vs. isolated remnant trees in pastures at different study regions, indicating vegetation type (VT), elevational range (ER), annual precipitation (AP), and references in chronological order; Table S6: Comparison of epiphyte species richness reduction in old-growth forest vs. disturbed, managed or selectively logged forests at different study regions, indicating vegetation type (VT), elevational range (ER), annual precipitation (AP), and references in chronological order.
