# Supplementary material for: Impact of Land-Use Change on Vascular Epiphytes: A Review
Source: Plants (Basel). 2025 Apr 11;14(8):1188. doi: 10.3390/plants14081188 (PMC12030193; doi:10.3390/plants14081188)
Supplement: Supplementary file 1 [file plants-14-01188-s001.zip › plants-3536668-supplementary.pdf]

Supplementary Materials

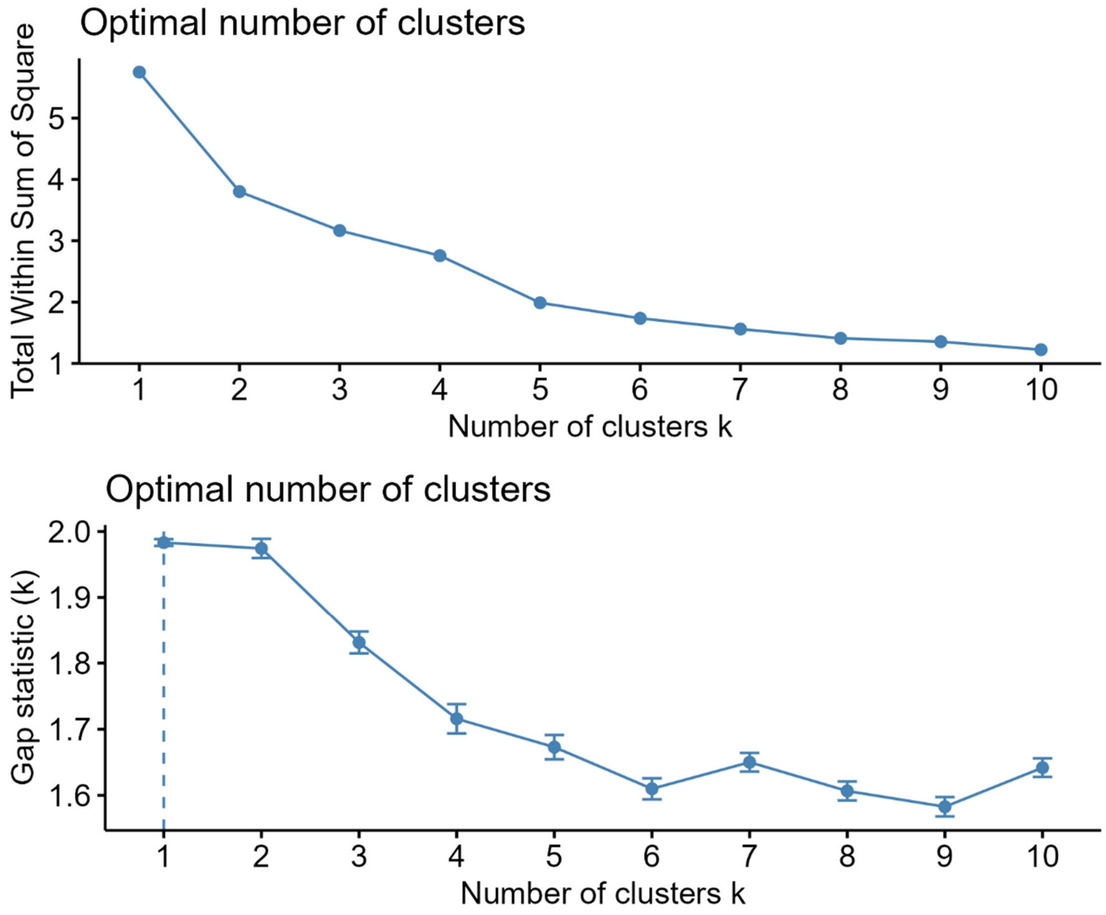

Figure S1. Visual assessment of the different methods for determining the optimal number of clusters for k-means, we used two types: total within sum of square or “wws” (upper graph) and gap statistics or "gap_stat" (lower graph) as a function of clusters. These methods include a direct method, consisting of optimizing a criterion, such as within cluster sums of squares, and statistical testing, consisting of comparing evidence against null hypothesis, respectively.

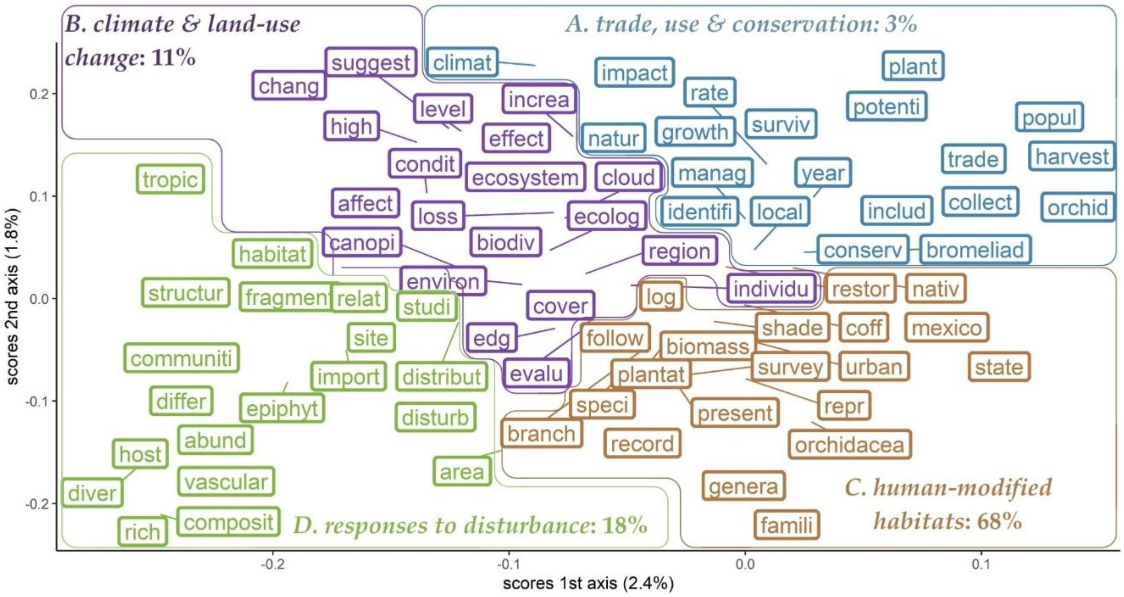

Figure S2. Four aspects of research A: *Trade, use and conservation*, B: *Climate and land-use change*, C: *Human-modified habitat*, D: *Responses to disturbance*. Here we show the 20 most frequent words per cluster derived from the overall matrix word (based on all abstracts), independently of their frequency or the number of papers represented in each cluster. Words varied in their frequency per cluster, these are depicted in Supplementary material, Figure S3.

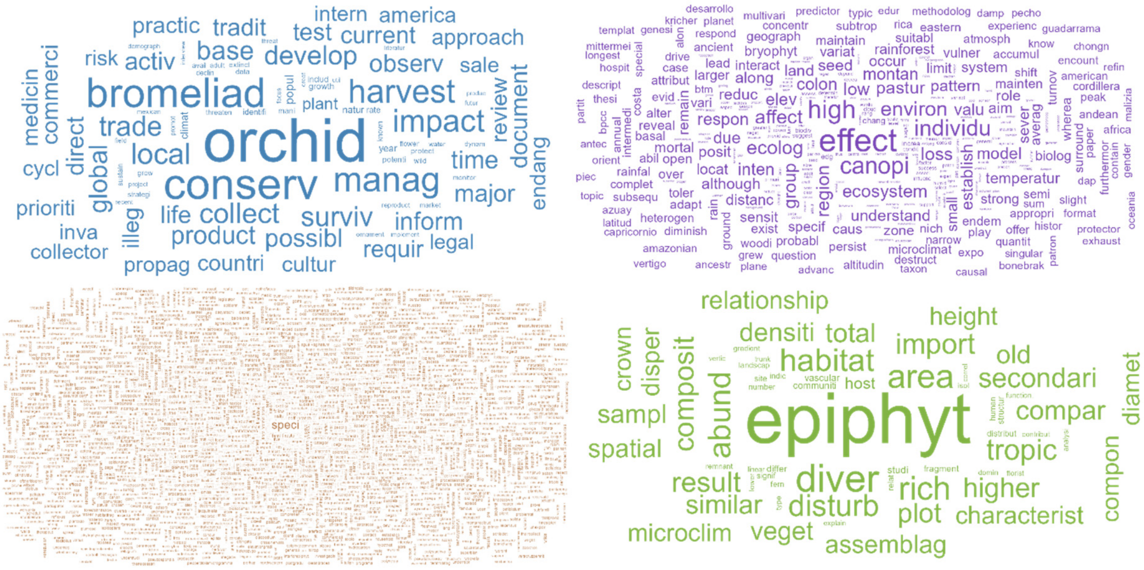

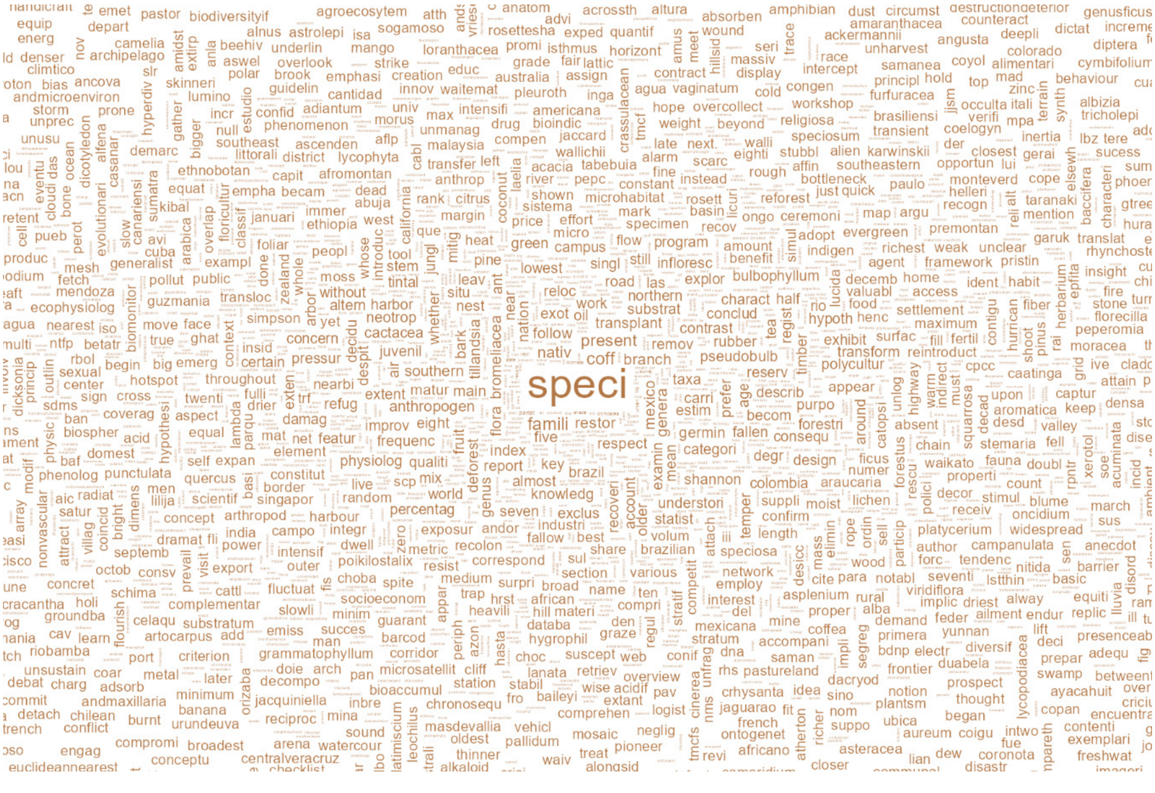

Figure S3. Word clouds of the four aspects of research (upper graph): *Trade, use and conservation* (upper left, word input from eight references, see Table S1), *Climate and land-use change*(upper right, word input from 33 references), *Human-modified*

habitats(lower left, word input from 195 references, an inset (lower graph) is provided for this category for the sake of visibility), *Responses to disturbance*(lower right, word input from 52 references). Word size reflects word frequency in relation to other words of the respective word cloud.

Table S1. List of references resulting in four clusters of the following aspects of research A: *Trade, use and conservation*, B: *Climate and land-use change*, C: *Human-modified habitats*, D: *Responses to disturbance*.

A	***Trade, use and conservation***
1	Elliott, D. D. (2014). Demography, wild harvest patterns and trade of culturally important species: priorities for management and conservation. Honolulu, HI, University of Hawaii at Manoa. PhD: 1-77.
2	Elliott, D. D. and T. Ticktin (2013). Epiphytic plants as NTFPs from the forest canopies: priorities for management and conservation. Treetops at Risk, Springer: 435-444.
3	Flores-Palacios, A. and S. Valencia-Diaz (2007). Local illegal trade reveals unknown diversity and involves a high species richness of wild vascular epiphytes. Biological Conservation 136(3): 372-387.
4	García-González, A., et al. (2017). Impact of different shade coffee management scenarios, on a population of *Oncidium poikilostalix* (Orchidaceae), in Soconusco, Chiapas, Mexico. Plant Ecology & Diversity 10(2-3): 185-196.
5	Hinsley, A., et al. (2018). A review of the trade in orchids and its implications for conservation. Botanical Journal of the Linnean Society 186(4): 435-455.
6	Mondragón Chaparro, D. and T. Ticktin (2011). Demographic effects of harvesting epiphytic bromeliads and an alternative approach to collection. Conservation Biology 25(4): 797-807.
7	Subedi, A., et al. (2013). Collection and trade of wild-harvested orchids in Nepal. Journal of ethnobiology and ethnomedicine 9(1): 64.
8	Ticktin, T., et al. (2020). Synthesis of wild orchid trade and demography provides new insight on conservation strategies. Conservation Letters 13(2): e12697.
**B**	***Climate and land-use change***
9	Abotsi, K. E., et al. (2020). Ecological drivers of pteridophyte diversity and distribution in Togo (West Africa). Ecological Indicators 108: 105741.
10	Abotsi, K. E., et al. (2020). Vulnerability of pteridophytes to climate change and implications for their conservation in Togo (west Africa). Plant Ecology and Evolution 153(1): 22-32.
11	Amici, A. A., et al. (2019). Contrasting effects of host tree isolation on population connectedness in two tropical epiphytic bromeliads. American Journal of Botany 106(12): 1602-1611.
12	Amici, A. A., et al. (2020). Differences in epiphyte biomass and community composition along landscape and within-crown spatial scales. Biotropica 52(1): 46-58.
13	Bianchi, J. S. and R. D. Kersten (2014). Edge effect on vascular epiphytes in a subtropical Atlantic Forest. Acta Botanica Brasilica 28(1): 120-126.

14	Cach-Perez, M. J., et al. (2014). Susceptibility of epiphytic bromeliads to climate change. Botanical Sciences 92(2): 157-168.
15	Carvajal-Hernández, C. I., et al. (2017). Conservation value of disturbed and secondary forests for ferns and lycophytes along an elevational gradient in Mexico. Applied Vegetation Science 20(4): 662-672.
16	Dias-Terceiro, R. G., et al. (2015). Edge effect on vascular epiphytic composition in a fragment of Atlantic Forest in northeastern Brazil. Acta Botanica Brasilica 29(2): 270-273.
17	Einzmann, H. J. R., et al. (2022). The impact of a severe El Nino event on vascular epiphytes in lowland Panama. Diversity 14(5): 325.
18	Einzmann, H. J. R. and G. Zotz (2017). No signs of saturation: long-term dynamics of vascular epiphyte communities in a human-modified landscape. Biodiversity and Conservation 26(6): 1393-1410.
19	Foster, P. (2001). The potential negative impacts of global climate change on tropical montane cloud forests. Earth-Science Reviews 55(1-2): 73-106.
20	Gabriel y Galán, J. M., et al. (2018). Germination fitness of two temperate epiphytic ferns shifts under increasing temperatures and forest fragmentation. PLoS One 13(5): e0197110.
21	Guzmán-Jacob, V., et al. (2020). Effects of forest-use intensity on vascular epiphyte diversity along an elevational gradient. Diversity and Distributions 26(1): 4-15.
22	Hsu, R. C. C., et al. (2012). Simulating climate change impacts on forests and associated vascular epiphytes in a subtropical island of East Asia. Diversity and Distributions 18(4): 334-347.
23	Hsu, R. C. C. and J. H. D. Wolf (2013). A novel approach to simulate climate change impacts on vascular epiphytes: case study in Taiwan. Treetops at Risk. M. Lowman, Devy, S and T. Ganesh, Springer: 123-130.
24	Jadán Maza, A. O. (2023). Ecological assembly of woody plants and vascular epiphytes: Floral composition, diversity and structure in tropical mountain forests, Universidad Rey Juan Carlos. Thesis. Ecuador.
25	Köster, N., et al. (2013). Range size and climatic niche correlate with the vulnerability of epiphytes to human land use in the tropics. Journal of Biogeography 40(5): 963-976.
26	Ma, W., et al. (2008). Edge effects on epiphytes in montane moist evergreen broad-leaved forest. Biodiversity Science 16(3): 245-254.
27	Nadkarni, N. M. and R. Solano (2002). Potential effects of climate change on canopy communities in a tropical cloud forest: an experimental approach. Oecologia 131(4): 580-586.
28	Nadkarni, N. M. (2023). Complex consequences of disturbance on canopy plant communities of world forests: a review and synthesis. New Phytologist, Wiley Online Library. 240: 1366-1380.
29	Parra Sánchez, E., et al. (2016). Edge influence on diversity of orchids in Andean cloud forests. Forests 7(3): 63.
30	Parra-Sanchez, E. and C. Banks-Leite (2020). The magnitude and extent of edge effects on vascular epiphytes across the Brazilian Atlantic Forest. Scientific Reports 10(1): 18847.

31	Parra-Sanchez, E. and D. P. Edwards (2024). Spatial extent predicts Andean epiphyte biodiversity responses to habitat loss and fragmentation across human-modified landscapes. Journal of Biogeography 51: 1315-1327.
32	Petter, G., et al. (2021). Agent-based modeling of the effects of forest dynamics, selective logging, and fragment size on epiphyte communities. Ecology and Evolution 11(6): 2937-2951.
33	Pouteau, R., et al. (2016). Fern species richness and abundance are indicators of climate change on high-elevation islands: evidence from an elevational gradient on Tahiti (French Polynesia). Climatic Change 138(1-2): 143-156.
34	Rapp, J. M. and M. R. Silman (2014). Epiphyte response to drought and experimental warming in an Andean cloud forest. F1000Res 3: 7.
35	Reyes-Chávez, J., et al. (2021). Nowhere to escape–Diversity and community composition of ferns and lycophytes on the highest mountain in Honduras. Journal of Tropical Ecology 37(2): 72-81.
36	Richards, J. H. (2020). Assessing the strength of climate and land-use influences on montane epiphyte communities. Conservation Biology 35(5): 1496-1506.
37	Sagar, R. and M. S. Devy (2022). The impact of anthropogenic disturbance to the canopy microclimate of tropical forests in the Southern Western Ghats, India. Frontiers in Forests and Global Change 5.
38	Shashidhar, K. S. and A. N. A. Kumar (2009). Effect of climate change on orchids and their conservation strategies. Indian Forester 135(8): 1039.
39	Werner, F. A. (2011). Reduced growth and survival of vascular epiphytes on isolated remnant trees in a recent tropical montane forest clear-cut. Basic and Applied Ecology 12(2): 172-181.
40	Zotz, G. and M. Y. Bader (2009). Epiphytic plants in a changing world-global: change effects on vascular and non-vascular epiphytes. Progress in botany. U. Lüttge, W. Beyschlag, B. Büdel and D. Francis. Berlin, Germany, Springer: 147-170.
**C**	***Human-modified habitats***
41	Acebey, A., et al. (2010). Ecoregional distribution of potentially useful species of Araceae and Bromeliaceae as non-timber forest products in Bolivia. Biodiversity and Conservation 19(9): 2553-2564.
42	Ackerman, J. (2007). Invasive orchids: weeds we hate to love? Lankesteriana 7: 19-21.
43	Adhikari, Y. P., et al. (2012). Micro-site conditions of epiphytic orchids in a human impact gradient in Kathmandu valley, Nepal. Journal of Mountain Science 9(3): 331-342.
44	Adhikari, Y. P., et al. (2015). Sustainable conservation perspectives for epiphytic orchids in the Central Himalayas, Nepal. Applied Ecology and Environmental Research 13(3): 753-767.
45	Adhikari, Y. P., et al. (2012). Host tree utilization by epiphytic orchids in different land-use intensities in Kathmandu Valley, Nepal. Plant Ecology 213(9): 1393-1412.

46	Aguirre, A., et al. (2010). Fate of epiphytes on phorophytes with different architectural characteristics along the perturbation gradient of *Sabal mexicana* forests in Veracruz, Mexico. Journal of Vegetation Science 21(1): 6-15.
47	Alanís Méndez, J. L., et al. (2007). Contributions to the knowledge of epiphytes (Bromeliaceae, Cactaceae and Orchidaceae) in two types of vegetation in the municipality of Pánuco, Veracruz, Mexico. Revista Científica U. D. O. Agrícola 7(1): 160-174.
48	Alex, A., et al. (2021). Diversity and phorophyte preference of vascular epiphytic flora on avenues within the University of Port Harcourt, Nigeria. Journal of Forest and Environmental Science. 37: 217-225.
49	Aoki-Gonçalves, F., et al. (2023). Urban epiphytes: Bromeliad diversity in a green cover gradient across a Neotropical streetscape. Urban Forestry & Urban Greening, Elsevier. 83: 127901.
50	Alvim, F. S., et al. (2021). Are vascular epiphytes in urban green areas subject to the homogenization of biodiversity? A case study in the Brazilian Atlantic Forest. Urban Ecosystems 24(4): 701-713.
51	Alvim, F. S., et al. (2020). Diversity of vascular epiphytes in urban green areas of Juiz de Fora, Minas Gerais, Brazil. Floresta E Ambiente 27(2): e20190116.
52	Araya, D. F. (2013). Response of vascular epiphytes of Nothofagus dombeyi (Nothofagaceae) to landscape fragmentation; conservation and environmental education proposal in the urban forest canopy of Valdivia, Chile. Valdiva, Chile, Universidad Austral de Chile. Ingeniero en Conservación de Recursos Naturales: 67.
53	Arias Chumbi, R. E. (2014). Response to selective logging and climate change of understory vascular epiphytes in a cloud forest in southern Ecuador. Cuenca, Ecuador, Universidad del Azuay. Biólogo del Medio Ambiente: 1-38.
54	Armenta-Montero, S., et al. (2015). Distribution and conservation status of *Phlegmariurus* (Lycopodiaceae) in the state of Veracruz, Mexico. Tropical Conservation Science 8(1): 114-137.
55	Baltazar-Bernal, O., et al. (2024). Orchid diversity (Orchidaceae) in two urban sites in the state of Veracruz, Mexico. Agrociencia: 1-11.
56	Barbosa, D. E. F., et al. (2020). The importance of heterogeneity of habitats for the species richness of vascular epiphytes in remnants of Brazilian montane seasonal semideciduous forest. Edinburgh Journal of Botany 77(1): 99-118.
57	Bataghin, F. A., et al. (2008). Edge effect in vascular epiphytes in a mixed ombrophilous forest, Rio Grande do Sul, Brazil. O Mundo da Saúde 32(3): 329-338.
58	Bautista, L. J., et al. (2014). Impact of silvicultural methods on vascular epiphytes (ferns, bromeliads and orchids) in a temperate forest in Oaxaca, Mexico. Forest Ecology and Management 329: 10-20.
59	Becker, D. F. P., et al. (2017). Richness, coverage and concentration of heavy metals in vascular epiphytes along an urbanization gradient. Science of The Total Environment 584-585: 48-54.

60	Beeretz, L. (2015). Diversity of vascular epiphytes in jungle rubber along a distance gradient to Bukit Duabelas National Park in Sumatra (Indonesia). Göttingen, Germany, University of Göttingen. MSc: 1-73.
61	Benavides, A. M., et al. (2006). Recovery and succession of epiphytes in upper Amazonian fallows. Journal of Tropical Ecology 22(6): 705-717.
62	Bennett, B. C. (1995). Ethnobotany and economic botany of epiphytes, lianas, and other host-dependent plants: an overview. Forest canopies. M. D. Lowman and N. M. Nadkarni. San Diego, California, Academic Press: 547-586.
63	Benzing, D. H. (1998). Vulnerabilities of tropical forests to climate change: the significance of resident epiphytes. Potential impacts of climate change on tropical forest ecosystems. A. Markham. Dordrecht, Springer: 379-400.
64	Bernardi, S. and J. C. Budke (2010). Structure of vascular epiphytes communities and the edge-effect relationships in a transitional area of seasonal semideciduous forest and rain mixed forest. Floresta 40(1): 81-92.
65	Besi, E. E., et al. (2019). Orchid diversity in anthropogenic-induced degraded tropical rainforest, an extrapolation towards conservation. Lankesteriana 19(2): 107-124.
66	Bhatt, A., et al. (2015). Epiphyte diversity on host trees in an urban environment, eThekwini Municipal Area, South Africa. New Zealand Journal of Botany 53(1): 24-37.
67	Boelter, C. R., et al. (2011). Exotic tree monocultures play a limited role in the conservation of Atlantic Forest epiphytes. Biodiversity and Conservation 20(6): 1255-1272.
68	Böhnert, T., et al. (2016). Effects of land-use change on vascular epiphyte diversity in Sumatra (Indonesia). Biological Conservation 202: 20-29.
69	Bonnet, A. and M. H. d. Queiroz (2006). Vertical stratification of epiphytic bromeliads on different stages of secondary succession of Atlantic Rainforest, in Santa Catarina Island, Santa Catarina, Brazil. Brazilian Journal of Botany 29(2): 217-228.
70	Borrero, H., et al. (2023). Populations of a tropical epiphytic orchid are destabilized in its peripheral range by hurricane and an exotic herbivore. Ecosphere, Wiley Online Library. 14: e4355.
71	Brighigna, L., et al. (2002). The use of tropical bromeliads (*Tillandsia* spp.) for monitoring atmospheric pollution in the town of Florence, Italy. Revista de Biologia Tropical 50(2): 577-584.
72	Bryan, C. L. (2011). Ecology of vascular epiphytes in urban forests with special reference to the shrub epiphyte Griselinia lucida. Hamilton, New Zealand, University of Waikato. MSc.
73	Carmona-Higuita, M. J., et al. (2024). Conservation status of vascular epiphytes in the neotropics. Biodiversity and Conservation 33: 51-71.
74	Carvajal-Hernández, C. I. and T. Krömer (2015). Richness and distribution of ferns and lycophytes in the altitudinal gradient of the Cofre de Perote, central Veracruz, Mexico. Botanical Sciences 93(3): 601-614.

75	Cascante-Marín, A., et al. (2009). Dispersal limitation in epiphytic bromeliad communities in a Costa Rican fragmented montane landscape. Journal of Tropical Ecology 25(1): 63-73.
76	Cerón Martínez, C. E. and C. I. Tello Reyes (2021). Epifitas de *Phoenix canariensis* Chabaud (Arecaceae) en cinco localidades Sudamericanas. Cinchonia. 16: 197-216.
77	Chaves, C. J. N., et al. (2018). How are endemic and widely distributed bromeliads responding to warming temperatures? A case study in a Brazilian hotspot. Flora 238: 110-118.
78	Chen, Q., et al. (2019). Obligate to facultative shift of two epiphytic *Lepisorus* species during subtropical forest degradation: Insights from functional traits. Forest Ecology and Management 435: 66-76.
79	Chinsamy, M., et al. (2011). The ethnobotany of South African medicinal orchids. South African Journal of Botany 77(1): 2-9.
80	Córdova, J. and R. F. Del Castillo (2001). Changes in epiphyte cover in three chronosequences in a tropical montane cloud forest in Mexico. Life Forms and Dynamics in Tropical Forests. G. Gottsberger and S. Liede. Stuttgart, Germany, J. Cramer in der Gebrüder Borntraeger Verlagsbuchhandlung: 79-94.
81	Cruz-Angón, A., et al. (2009). The contribution of epiphytes to the abundance and species richness of canopy insects in a Mexican coffee plantation. Journal of Tropical Ecology 25(5): 453-463.
82	Cruz-Fernández, Q. T., et al. (2011). Is orchid species richness and abundance related to the conservation status of oak forest? Plant Ecology 212(7): 1091-1099.
83	Cruz-Garcia, G., et al. (2015). The wild orchid trade in a Mexican local market: Diversity and economics. Economic Botany 69(4): 291-305.
84	D'Cunha, P. J. and P. V. Gowda (2013). Epiphyte diversity on avenue trees of national and state highways of Udupi district, Karnataka, India. International Research Journal of Biological Sciences. 2: 30-39.
85	De Beenhouwer, M., et al. (2015). Management intensification in Ethiopian coffee forests is associated with crown habitat contraction and loss of specialized epiphytic orchid species. Basic and Applied Ecology 16(7): 592-600.
86	de Carvalho, A. J. A., et al. (2020). Vascular epiphytes on licuri palms (*Syagrus coronata* (Mart.) Becc.) in a toposequence: Caatinga conservation indicator species. Brazilian Journal of Botany 43(4): 1061-1075.
87	de Oliveira Alves, M. E., et al. (2014). A survey of vascular epiphyte species of the urban area of Palmeira das Missoes, RS, Brazil. Ciência e Natura 36(3): 268-276.
88	de Oliveira, L. C., et al. (2013). Vascular epiphytic component in an urban forest fragment in Criciuma, Santa Catarina, Brazil. Biotemas 26(2): 33-44.
89	Del Castillo, R. F., et al. (2013). Possible combined effects of climate change, deforestation, and harvesting on the epiphyte *Catopsis compacta*: a multidisciplinary approach. Ecology and Evolution 3(11): 3935-3946.
90	Duarte, M. M. and S. Gandolfi (2017). Diversifying growth forms in tropical forest restoration: Enrichment with vascular epiphytes. Forest Ecology and Management 401: 89-98.

91	Einzmann, H. J. R. and G. Zotz (2017). Dispersal and establishment of vascular epiphytes in human-modified landscapes. AoB Plants 9(6): plx052.
92	Elias, N. (2008). Road edge effect on forest canopy structure and epiphyte biodiversity in a tropical mountainous rainforest Nyungwe National Park, Rwanda. Enschede, NL, International Institute for Geo-Information Science and Earth Observation. MSc.
93	Emeterio-Lara, A., et al. (2021). Does extraction of orchids affect their population structure? Evidence from populations of Laelia autumnalis (Orchidaceae). Forest Ecology and Management 480: 118667.
94	Emeterio-Lara, A., et al. (2021). Is pseudobulb harvest a sustainable management strategy in wild orchid populations? An experiment with *Laelia autumnalis*. Forest Ecology and Management 491: 119205.
95	Espejo Serna, A., et al. (2005). Orchids from coffee-plantations in Mexico: an alternative for the sustainable use of tropical ecosystems. Revista de Biologia Tropical 53(1-2): 73-84.
96	Fabricante, J. R., et al. (2006). Vascular epiphyte component occuring in urban trees. Cerne 12(4): 399-405.
97	Fayle, T. M., et al. (2010). Oil palm expansion into rain forest greatly reduces ant biodiversity in canopy, epiphytes and leaf-litter. Basic and Applied Ecology 11(4): 337-345.
98	Fernandez Barrancos, E. P., et al. (2017). Tank bromeliad transplants as an enrichment strategy in southern Costa Rica. Restoration Ecology 25(4): 569-576.
99	Furtado, S. G. and L. M. Neto (2015). Diversity of vascular epiphytes in urban environment: a case study in a biodiversity hotspot, the Brazilian Atlantic Forest. Ces Revista 29(2): 82-101.
100	García-Franco, J. G. and T. Toledo (2008). Vascular epiphytes: bromeliads and orchids. Agroecosistemas cafetaleros de Veracruz Biodiversidad manejo y conservación. Xalapa, Mexico, Instituto de Ecología, A. C.: 69-82.
101	Garcia-Gonzalez, A., et al. (2021). Assemblage of vascular epiphytes associated to seasonally inundated forest in the Southeastern Mexico: Challenges for its conservation. Global Ecology and Conservation 25: e01404.
102	Goodall, K. E., et al. (2015). Shade tree diversity, carbon sequestration, and epiphyte presence in coffee agroecosystems: A decade of smallholder management in San Ramon, Nicaragua. Agriculture Ecosystems & Environment 199: 200-206.
103	Gutiérrez Pérez, N. Y. (2014). Diversity of vascular epiphytes in disturbed forests of different ages in the San Francisco River Valley of southern Ecuador. Tacna, Peru, Universidad Nacional Jorge Basadre Grohmann. Escuela Académico. Biólogo Microbiólogo: 71.
104	Haeckel, I. B. (2008). The arco floral: Ethnobotany of *Tillandsia* and *Dasylirion* spp. in a Mexican religious adornment. Economic Botany 62(1): 90-95.
105	Hietz, P. (2005). Conservation of vascular epiphyte diversity in Mexican coffee plantations. Conservation Biology 19(2): 391-399.

106	Hietz, P., et al. (2012). Germination of epiphytic bromeliads in forests and coffee plantations: Microclimate and substrate effects. Biotropica 44(2): 197-204.
107	Hornung-Leoni, C. T. (2011). Advances on ethnobotanical uses of Bromeliaceae in Latin America. Boletin Latinoamericano Y Del Caribe De Plantas Medicinales Y Aromaticas 10(4): 297-314.
108	Hossain, M. M. (2011). Therapeutic orchids: traditional uses and recent advances—an overview. Fitoterapia 82(2): 102-140.
109	Hundera, K., et al. (2013). Both forest fragmentation and coffee cultivation negatively affect epiphytic orchid diversity in Ethiopian moist evergreen Afromontane forests. Biological Conservation 159: 285-291.
110	Hylander, K. and S. Nemomissa (2008). Home garden coffee as a repository of epiphyte biodiversity in Ethiopia. Frontiers in Ecology and the Environment 6(10): 524-528.
111	Hylander, K. and S. Nemomissa (2017). Waiving the extinction debt: Can shade from coffee prevent extinctions of epiphytic plants from isolated trees? Diversity and Distributions 23(8): 888-897.
112	Hylander, K., et al. (2013). Edge effects on understory epiphytic ferns and epiphyllous bryophytes in moist afromontane forests of Ethiopia. Polish Botanical Journal 58(2): 555-563.
113	Izuddin, M. and E. L. Webb (2015). The influence of tree architecture, forest remnants, and dispersal syndrome on roadside epiphyte diversity in a highly urbanized tropical environment. Biodiversity and Conservation 24(8): 2063-2077.
114	Izuddin, M., et al. (2018). Specific niche requirements drive long-term survival and growth of translocated epiphytic orchids in an urbanised tropical landscape. Urban Ecosystems 21(3): 531-540.
115	Jiménez-López, D. A., et al. (2019). Wild orchids traded in a traditional market in Chiapas, Mexico. Botanical Sciences 97(4): 691-700.
116	Jiménez-López, D. A., et al. (2019). Ceremonial use of bromeliads and other vascular epiphytes in cemeteries of two indigenous communities of Las Margaritas, Chiapas, Mexico. Economic Botany 73(1): 127-132.
117	Jiménez-Orozco, C., et al. (2019). Characterization of the vascular epiphytic flora of Iberoamerica Park, Santo Domingo, Dominican Republic. Ciencia Ambiente y Clima 2(1): 23-33.
118	Kartzinel, T. R., et al. (2013). Critical importance of large native trees for conservation of a rare Neotropical epiphyte. Journal of Ecology 101(6): 1429-1438.
119	Kersten, R. d. A. and Y. S. Kuniyoshi (2009). Forest conservation on the Iguaçu river basin, Paraná-evaluation of the vascular epiphyte community in different seres. Floresta 39(1): 51-66.
120	Kimpouni, V., et al. (2017). Floristic diversity of epiphytes and vascular hemiparasites of the urban forest ecosystem of Brazzaville, Congo. Journal of Applied Biosciences 117: 11704-11719.
121	Krömer, T., et al. (2018). Use of epiphytic plants: implications for their conservation and sustainable management. De la recolección a los agroecosistemas soberanía alimentaria y conservación de la

	biodiversidad. Silva, E. Rivera, V. Martínez Valdés, M. Lascurain and E. Rodríguez Luna. Xalapa, Veracruz, Mexico, Universidad Veracruzana: 175-196.
122	Krömer, T. and S. R. Gradstein (2003). Species richness of vascular epiphytes in two primary forests and fallows in the Bolivian Andes. Selbyana 24(2): 190-195.
123	Lippert, A. P. U., et al. (2022). Edge effect on vascular epiphytes in a subtropical Atlantic Forest fragment. Journal of Environmental Analysis and Progress 7(3): 135-149.
124	Liu, H., et al. (2014). Eat your orchid and have it too: a potentially new conservation formula for Chinese epiphytic medicinal orchids. Biodiversity and Conservation 23(5): 1215-1228.
125	Liu, Q., et al. (2015). Orchid conservation in the biodiversity hotspot of southwestern China. Conservation Biology 29(6): 1563-1572.
126	López-Trabanco, P. J. and S. Orta-Pozo (2012). Human disturbances over the epiphyte orchids in the Biosphere Reserve ¨Sierra del Rosario". Avances 14(4): 420-433.
127	Lugo, A. E. and F. N. Scatena (1992). Epiphytes and climate change research in the Caribbean: a proposal. Selbyana 13: 123-130.
128	Madison, M. (1979). Distribution of epiphytes in a rubber plantation in Sarawak. Selbyana 5(2): 207-213.
129	Magrach, A., et al. (2012). Internal habitat quality determines the effects of fragmentation on austral forest climbing and epiphytic angiosperms. PLoS One 7(10): e48743.
130	Magrach, A., et al. (2014). Edge effects shape the spatial distribution of lianas and epiphytic ferns in Australian tropical rain forest fragments. Applied Vegetation Science 17(4): 754-764.
131	Martínez-Meléndez, N., et al. (2022). Importance of *Quercus* spp. for diversity and biomass of vascular epiphytes in a managed pine-oak forest in Southern Mexico. Forest Ecosystems 9: 100034.
132	Martínez-Meléndez, N., et al. (2018). Epiphyte diversity in a pine-oak forest with silvicultural activities in Chiapas, Mexico. Desde el Herbario Cicy 10: 160-167.
133	Martins, P. L. S. S., et al. (2020). Could epiphytes be xenophobic? Evaluating the use of native versus exotic phorophytes by the vascular epiphytic community in an urban environment. Community Ecology 21(1): 91-101.
134	Mehltreter, K. (2008). Ferns. Agroecosistemas cafetaleros de Veracruz biodiversidad manejo y conservación. México D. F., Mexico, Instituto de Ecología A. C. Instituto Nacional de Ecología Secretaría de Medio Ambiente y Recursos Naturales: 83-93.
135	Merwin, M. C., et al. (2003). The influence of host tree species on the distribution of epiphytic bromeliads in experimental monospecific plantations, La Selva, Costa Rica. Biotropica 35(1): 37-47.
136	Mondragón, D. and M. P. Mora-Flores (2023). First steps to study the demography of vascular epiphytes in cities. Brazilian Journal of Biology, SciELO Brasil. 84: e270998.

137	Mondragón, D., et al. (2016). Prioritizing the conservation of epiphytic bromeliads using ethnobotanical information from a traditional Mexican market. Economic Botany 70(1): 29-36.
138	Mondragón, D., et al. (2009). Epiphyte diversity on coffee bushes: A management question? Journal of Sustainable Agriculture 33(7): 703-715.
139	Mondragón, D. and D. M. Villa-Guzmán (2008). Ethnobotanical study of epiphytic bromeliads in Santa Catarina Ixtepeji community, Oaxaca, Mexico. Polibotánica 26(26): 175-191.
140	Morales-Linares, J., et al. (2020). The role of shaded cocoa plantations in the maintenance of epiphytic orchids and their interactions with phorophytes. Journal of Plant Ecology 13(1): 27-35.
141	Moreno-Barreto, E. and A. M. Medina-Sánchez (2024). Floristic and functional characteristics of the vascular epiphytes associated with the palm trees of the *Phoenix* L. genus in Bogotá, Colombia. Colombian biota. 25:e1155.
142	Mucunguzi, P. (2007). Diversity and distribution of epiphytic ferns in Kibale National Park, Uganda. Selbyana 28(2): 154-160.
143	Mucunguzi, P. (2007). Diversity and distribution of hemi-epiphytes and facultative herbaceous epiphytes in Kibale National Park, Uganda. African Journal of Ecology 45(s1): 57-64.
144	Mucunguzi, P. (2008). Diversity and distribution of epiphytic orchids in Kibale National Park, Uganda. Selbyana 29(2): 217-225.
145	Nadège, M. T., et al. (2017). Community of vascular epiphytes on some phorophytes in the Babadjou Subdivision (Western Cameroon): Case of Bamelo. Journal of Sustainable Forestry 36(1): 65-75.
146	Nadkarni, N. M. (2000). Colonization of stripped branch surfaces by epiphytes in a lower montane cloud forest, Monteverde, Costa Rica. Biotropica 32(2): 358-363.
147	Nadkarni, N. M. and W. A. Haber (2009). Canopy seed banks as time capsules of biodiversity in pasture-remnant tree crowns. Conservation Biology 23(5): 1117-1126.
148	Negrelle, R. R. B., et al. (2012). Bromeliad ornamental species: conservation issues and challenges related to commercialization. Acta Scientiarum Biological Sciences 34(1): 91-100.
149	Nfornkah, B. N., et al. (2019). Vascular epiphytes loss in exploited trees of the semi deciduous managed forest of Ndelele, East Cameroon. Journal of Sustainable Forestry 38(7): 670-685.
150	Nöske, N. M., et al. (2008). Disturbance effects on diversity of epiphytes and moths in a montane forest in Ecuador. Basic and Applied Ecology 9(1): 4-12.
151	Obermüller, F. A., et al. (2012). Epiphytic (including hemiepiphytes) diversity in three timber species in the southwestern Amazon, Brazil. Biodiversity and Conservation 21(2): 565-575.
152	Oishi, Y. and H. Doei (2015). Changes in epiphyte diversity in declining forests: implications for conservation and restoration. Landscape and Ecological Engineering 11(2): 283-291.

153	Orozco Ávila, J., et al. (2017). Estimation of the transfer of vascular epiphytes, as a conservation strategy in the municipality of Aguazul, Casanare, Colombia. Revista de investigación agraria y ambiental 8(1): 27-37.
154	Orozco-Ibarrola, O., et al. (2021). Sustainable harvesting and conservation of *Laelia furfuracea*, a rare epiphytic orchid from Oaxaca, Mexico. Biotropica 53(1): 142-151.
155	Osie, M., et al. (2022). Habitat fragmentation effects on vascular epiphytes diversity in Kafa biosphere reserve and nearby coffee agroecosystem, southwestern Ethiopia. Tropical Ecology: 1-11.
156	Padilha, P. T., et al. (2017). Vascular epiphytes respond to successional stages and microhabitat variations in a subtropical forest in southern Brazil. Brazilian Journal of Botany 40(4): 897-905.
157	Padmawathe, R., et al. (2004). Effects of selective logging on vascular epiphyte diversity in a moist lowland forest of Eastern Himalaya, India. Biological Conservation 119(1): 81-92.
158	Parra Sánchez, E. (2018). The effects of human disturbance on vascular epiphyte in the Brazilian Atlantic Forest. London, UK, Imperial College London. Doctor of Philosophy (PhD): 174.
159	Parra-Sanchez, E. and C. Banks-Leite (2022). Value of human-modified forests for the conservation of canopy epiphytes. Biotropica 54(4): 958-968.
160	Parra-Tabla, V., et al. (2000). Female and male pollination success of *Oncidium ascendens* Lindey (Orchidaceae) in two contrasting habitat patches: forest vs agricultural field. Biological Conservation 94(3): 335-340.
161	Pedroso-De-Moraes, C., et al. (2015). Edge effect on orchids of a fragment of semi-deciduous seasonal forest in the Southeast of Brazil. Iheringia Serie Botanica 70(1): 115-127.
162	Pérez-Ginez, I., et al. (2022). Effect of forestry research on the diversity of vascular epiphytes in a fragment of pine forest in the municipality of Tequila, Veracruz, Mexico. Proyecta Revista Científica: 82-103.
163	Pérez-Peña, A. and T. Krömer (2017). What can secondary forests and citrus plantations contribute to the conservation of vascular epiphytes in Los Tuxtlas, Veracruz? Avances y perspectivas en la investigación de los bosques tropicales y sus alrededores la región de Los Tuxtlas. Reynoso, V. H, R. I. Coates and M. D. L. V. Vázquez. Ciudad de México, Mexico, Instituto de Biología, Universidad Nacional Autónoma de México: 569-580.
164	Pett-Ridge, J. and W. L. Silver (2002). Survival, growth, and ecosystem dynamics of displaced bromeliads in a montane tropical forest. Biotropica 34(2): 211-224.
165	Pie, M. R., et al. (2022). Fern and lycophyte niche displacement under predicted climate change in Honduras. Plant Ecology 223(6): 613-625.
166	Pincheira-Ulbrich, J., et al. (2019). Consequences of swamp forest fragmentation on assemblages of vascular epiphytes and climbing plants: Evaluation of the metacommunity structure. Ecology and Evolution 8(23): 11785-11798.

167	Poltz, K. and G. Zotz (2011). Vascular epiphytes on isolated pasture trees along a rainfall gradient in the lowlands of Panama. Biotropica 43(2): 165-172.
168	Porembski, S. and N. Biedinger (2001). Epiphytic ferns for sale: Influence of commercial plant collection on the frequency of *Platycerium stemaria* (Polypodiaceae) in coconut plantations on the southeastern Ivory Coast. Plant Biology 3(1): 72-76.
169	Prescott, G. W., et al. (2015). Retaining biodiversity in intensive farmland: epiphyte removal in oil palm plantations does not affect yield. Ecology and Evolution 5(10): 1944-1954.
170	Quail, M. R., et al. (2023). Surrounded by concrete: genetic isolation of *Tillandsia recurvata* L. in an urban landscape in southeastern Brazil. Botanical Journal of the Linnean Society. 203: 390-400.
171	Ranil, R. H. G., et al. (2015). Ornamental pteridophytes: an underexploited opportunity for the Sri Lankan floriculture industry. Journal of the National Science Foundation of Sri Lanka 43(4): 293-301.
172	Raventós, J., et al. (2018). Comparison of transient and asymptotic perturbation analyses of three epiphytic orchid species growing in coffee plantations in Mexico: effect on conservation decisions. Plant Ecology & Diversity 11(2): 133-145.
173	Read, M. (1989). Bromeliads threatened by trade. The Kew Magazine 6(1): 22-29.
174	Reiter, N., et al. (2016). Orchid re-introductions: an evaluation of success and ecological considerations using key comparative studies from Australia. Plant Ecology 217(1): 81-95.
175	Rendón-Aguilar, B., et al. (2017). Ethnobotany of lycophyta and polypodiophyta in priority terrestrial regions of Oaxaca, Mexico. American Fern Journal 107(4): 200-218.
176	Richards, J. H., et al. (2020). Tree longevity drives conservation value of shade coffee farms for vascular epiphytes. Agriculture Ecosystems & Environment 301: 107025.
177	Riefner Jr, R. E. and A. R. Smith (2019). New and noteworthy epiphytic ferns from the urban forests of Coastal Southern California, USA. Phytologia 101(1): 81-112.
178	Riofrío, L., et al. (2007). Spatial structure of *Pleurothallis*, *Masdevallia*, *Lepanthes* and *Epidendrum* epiphytic orchids in a fragment of montane cloud forest in South Ecuador. Lankesteriana 7(1-2): 102-106.
179	Riofrío, M., et al. (2023). Genetic diversity and structure in two epiphytic orchids from the montane forests of southern Ecuador: The role of overcollection on *Masdevallia rosea* in comparison with the widespread *Pleurothallis lilijae*. Plos one 18: e0290604.
180	Rodriguez, J. H., et al. (2011). Air quality biomonitoring in agricultural areas nearby to urban and industrial emission sources in Córdoba province, Argentina, employing the bioindicator *Tillandsia capillaris*. Ecological Indicators 11(6): 1673-1680.
181	Rogers, H. C. and B. D. Clarkson (2023). Epiphyte-host relationships of remnant and recombinant urban ecosystems in Hamilton, New Zealand: the importance of *Dicksonia squarrosa* (G. Forst.) Sw., whekî. New Zealand Journal of Botany, Taylor & Francis: 1-10.

182	Rojas-Méndez, K. J., et al. (2017). Massive extraction of the orchid *Laelia speciosa* (HBK) Schltr. for trading in local markets affect its population genetic structure in a fragmented landscape in central Mexico. Tropical Conservation Science 10: 1940082917693235.
183	Rooke-Devoy, T. H. (2023). Diversity and conservation of urban epiphytes in Central Auckland. Auckland, University of Auckland. Tesis. New Zealand.
184	Ruano Lajones, D. J., et al. (2023). Epífitas existentes en el bosque del Jardín Tropical en la Universidad Técnica Luis Vargas Torres. Revista Científica Arbitrada Multidisciplinaria PENTACIENCIAS. 5: 337-353.
185	Ruas, R. d. B., et al. (2024). A bromeliad living in the city: a case of a native species resilient to urbanization in South Brazil. Botanical Journal of the Linnean Society. 205: 161-176.
186	Santana, L. D., et al. (2017). Diversity, vertical structure and floristic relationships of vascular epiphytes in an urban remnant of the Brazilian Atlantic Forest. Hoehnea 44: 123-138.
187	Saransig-León, H. I., et al. (2024). Conservation state of ferns and lycophytes in the Western Andes of Ecuador. Acta Biológica Colombiana. 29.
188	Scheffknecht, S., et al. (2010). Seedling establishment of epiphytic orchids in forests and coffee plantations in Central Veracruz, Mexico. Journal of Tropical Ecology 26(1): 93-102.
189	Scheffknecht, S., et al. (2012). Survival and growth of juvenile bromeliads in coffee plantations and forests in Central Veracruz, Mexico. Biotropica 44(3): 341-349.
190	Schmitt, J. L. and M. N. Goetz (2011). Species richness of ferns and lycophytes in an urban park in the Rio dos Sinos basin, Southern Brazil. Brazilian Journal of Biology 70(4 Suppl): 1161-1167.
191	Seshadri, K. S., et al. (2021). Persistent effects of historical selective logging on a vascular epiphyte assemblage in the forest canopy of the Western Ghats, India. Frontiers in Forests and Global Change 4: 727422.
192	Shaikh, S. D. and D. Meena (2011). Effects of mining on the diversity of the pteridophytes from the Western Ghats of Maharashtra (India). International Journal of Forest Usufructs Management 12(1): 57-61.
193	Sierra-Giraldo, J. A., et al. (2023). Collect, transport, relocate and monitor vascular epiphytes (Araceae, Bromeliaceae and Orchidaceae) in the Guavio region (Colombian Oriental Cordillera). Scientific Bulletin. Museum Center. Museo de Historia Natural, Universidad de Caldas. 27:33-51.
194	Silva, I. A., et al. (2011). Edge effects on fern community in an Atlantic Forest remnant of Rio Formoso, PE, Brazil. Brazilian Journal of Biology 71(2): 421-430.
195	Silvera, K. and E. Lasso (2016). Ecophysiology and crassulacean acid metabolism of tropical epiphytes. Tropical Tree Physiology. G. Goldstein and L. Santiago, Springer: 25-43.
196	Sinu, P. A., et al. (2011). Epiphytic orchid diversity in farmer-managed *Soppinabetta* forests of Western Ghats: implications for conservation. Current Science 101(10): 1337-1346.

197	Solano Gómez, R., et al. (2010). Plants used in the celebration of Holy Week in Zaachila, Oaxaca, Mexico. Polibotánica 29(29): 263-279.
198	Solis-Montero, L., et al. (2005). Shade-coffee plantations as refuges for tropical wild orchids in central Veracruz, Mexico. Conservation Biology 19(3): 908-916.
199	Solís-Montero, L., et al. (2019). Impact of moss and epiphyte removal on coffee production and implications for epiphyte conservation in shade coffee plantations in southeast Mexico. Agroecology and sustainable food systems 43(10): 1124-1144.
200	Sosa, V. and T. Platas (1998). Extinction and persistence of rare orchids in Veracruz, Mexico. Conservation Biology 12(2): 451-455.
201	Souza, V. D., et al. (2015). Richness and abundance of *Aechmea* and *Hohenbergia* (Bromeliaceae) in forest fragments and shade cocoa plantations in two contrasting landscapes in southern Bahia, Brazil. Tropical Conservation Science 8(1): 58-75.
202	Susan-Tepetlan, T. M., et al. (2015). Changes in the functional characteristics of vascular epiphytes of humid montane forest and secondary vegetation in the central region of Veracruz, Mexico. Botanical Sciences 93(1): 153-163.
203	Tamaki, V., et al. (2023). Propagation of two epiphytic Cactaceae for relocation to an urban protected area of the Atlantic Forest in São Paulo, Brazil. Rodriguésia 74: e01482021.
204	Toledo-Aceves, T., et al. (2012). Recolonization of vascular epiphytes in a shaded coffee agroecosystem. Applied Vegetation Science 15(1): 99-107.
205	Toledo-Aceves, T., et al. (2014). Bromeliad rain: An opportunity for cloud forest management. Forest Ecology and Management 329: 129-136.
206	Toledo-Aceves, T., et al. (2014). Potential impact of harvesting on the population dynamics of two epiphytic bromeliads. Acta Oecologica 59: 52-61.
207	Toledo-Aceves, T., et al. (2013). Benefits and costs of epiphyte management in shade coffee plantations. Agriculture Ecosystems & Environment 181: 149-156.
208	Trejo-Cruz, I. A., et al. (2021). Diversity of vascular epiphytes in remnant trees of the genus *Ficus* (Moraceae) in silvopastoral systems of southeastern Mexico. Acta Botanica Mexicana 128(128): e1827.
209	Triana-Moreno, L. A., et al. (2003). Vascular epiphytes as regeneration indicators of disturbed forests of the Colombian Amazon Region. Acta Biológica Colombiana 8(2): 31-42.
210	Turner, I. M., et al. (1994). A study of plant-species-extinction in Singapore - Lessons for the conservation of tropical biodiversity. Conservation Biology 8(3): 705-712.
211	Valencia Marin, A. (2013). Evaluation of the transfer of vascular epiphytes as a conservation strategy in the municipality of Aguazul, department of Casanare (preliminary study). Manizales, Colombia, Universidad de Manizales. Maestría en Desarrollo Sostenible y Medio Ambiente: 95.

212	Valenzuela Toledo, L. Y. (2023). Implement the retrieval, relocation maintenance and monitoring of epiphytes in the Miraflores RESERVOIR by applying the geographic information systems (GIS), Universidad de Manizales. Thesis. Caldas, Colombia.
213	Vandunné, H. J. F. (2002). Effects of the spatial distribution of trees, conspecific epiphytes and geomorphology on the distribution of epiphytic bromeliads in a secondary montane forest (Cordillera Central, Colombia). Journal of Tropical Ecology 18(2): 193-213.
214	Velázquez-Cárdenas, Y., et al. (2021). Do harvest practices of bromeliads and forest management in Sierra Norte of Oaxaca have a negative effect on their abundance and phorophyte preference? Ethnobiology Conservation 10: 1-19.
215	Velázquez-Juárez, Z. B., et al. (2023). Diversity and conservation of commercially grown orchards in Tenango, Puebla, Mexico. UVserva: Revista electrónica de la Coordinación Universitaria de Observatorios de la Universidad Veracruzana, Universidad Veracruzana: 120-137.
216	Verhoeven, K. J. F. and G. J. L. Beckers (1999). Canopy farming: an innovative strategy for the sustainable use of rain forests. Selbyana 20(1): 191-193.
217	Vermeulen, J. J., et al. (2014). Notes on *Bulbophyllum* (Dendrobiinae; Epidendroideae; Orchidaceae): two new species and the dilemmas of species discovery via illegal trade. Phytotaxa 184(1): 12-22.
218	Vieira Pacheco, J. R. and F. F. V. Antolin Barberena (2021). Floristic composition and community structure of epiphytic angiosperms in an urban forest fragment in the Eastern Amazon. Research in Ecology 3.
219	Wagner, K. and G. Zotz (2018). Epiphytic bromeliads in a changing world: The effect of elevated CO_2 and varying water supply on growth and nutrient relations. Plant Biology 20(3): 636-640.
220	Wallace, K. J. and B. D. Clarkson (2019). Urban forest restoration ecology: a review from Hamilton, New Zealand. Journal of the Royal Society of New Zealand 49(3): 347-369.
221	Wallace, K. J., et al. (2017). Exotic weeds and fluctuating microclimate can constrain native plant regeneration in urban forest restoration. Ecological Applications 27(4): 1268-1279.
222	Williams-Linera, G., et al. (1995). The fate of epiphytic orchids after fragmentation of a Mexican cloud forest. Selbyana 16(1): 36-40.
223	Winkler, M., et al. (2011). High gene flow in epiphytic ferns despite habitat loss and fragmentation. Conservation Genetics 12(6): 1411-1420.
224	Wolf, J. H. D. (2010). Embracing epiphytes in sustainable forest management: a pilot study from the Highlands of Chiapas, Mexico. Tropical montane cloud forests. New York, USA, Cambridge University Press: 652-658.
225	Wolf, J. H. D. and A. Flamenco-S (2006). Vascular epiphytes and their potential as a conservation tool in pine-oak forests of Chiapas, Mexico. Ecology and Conservation of Neotropical Montane Oak Forests. M. Kappelle, Springer: 375-391.

226	Wolf, J. H. D. and C. J. F. Konings (2001). Toward the sustainable harvesting of epiphytic bromeliads: a pilot study from the highlands of Chiapas, Mexico. Biological Conservation 101(1): 23-31.
227	Wu, J. and J. Brock (2023). The invasion of non-native epiphyte *Platycerium bifurcatum* in Auckland's urban forest canopy. New Zealand Journal of Ecology. 47.
228	Wu, S.-M., et al. (2024). Ancient tea gardens play important role on in situ conservation of epiphytic orchids in southwest Yunnan, China. Global Ecology and Conservation, Elsevier. 49: e02778.
229	Yam, T. W., et al. (2011). Conservation and reintroduction of native orchids of Singapore – the next phase. European Journal of Environmental Sciences. 1: 38-47.
230	Yañez, A., et al. (2021). An urban fern refugium: Municipal Ecological Reserve of Avellaneda (Eco Area) (Buenos Aires, Argentina). Blumea. 66: 227-235.
231	Zotz, G. (2016). Epiphytes and humans. Plants on Plants–The Biology of Vascular Epiphytes, Springer: 245-265.
232	Zotz, G., et al. (2023). CAM plants: their importance in epiphyte communities and prospects with global change. Annals of Botany, Oxford University Press US. 132: 685-698.
233	Zotz, G. and A. Cascante-Marín (2024). Life on the wire—plant growth on power lines in the Americas. Diversity. 16: 573.
**D**	***Responses to disturbance***
234	Acuna-Tarazona, M., et al. (2015). Post-stripping recolonization of vascular epiphytes in cloud-forest fragments in Mexico. Journal of Tropical Ecology 31(6): 499-508.
235	Adhikari, Y. P., et al. (2021). Vascular epiphyte diversity and host tree architecture in two forest management types in the Himalaya. Global Ecology and Conservation 27: e01544.
236	Barthlott, W., et al. (2001). Diversity and abundance of vascular epiphytes: a comparison of secondary vegetation and primary montane rain forest in the Venezuelan Andes. Plant Ecology 152(2): 145-156.
237	Bataghin, F. A., et al. (2012). Vascular epiphytes at the edge and interior of a semideciduous forest in Southeastern Brazil. Hoehnea 39(2): 235-245.
238	Cascante Marín, A., et al. (2008). Establishment of epiphytic bromeliads in successional tropical premontane forests in Costa Rica. Biotropica 40(4): 441-448.
239	Cascante-Marín, A., et al. (2006). Epiphytic bromeliad communities in secondary and mature forest in a tropical premontane area. Basic and Applied Ecology 7(6): 520-532.
240	Ceballos, S. J. (2020). Vascular epiphyte communities in secondary and mature forests of a subtropical montane area. Acta Oecologica 105: 103571.
241	Cuevas Reyes, P. and J. I. V. Gutiérrez (2012). Changes in the structure, composition and phenology of epiphytic plants under different stages of plant succession in a tropical dry forest. Biológicas 14(1): 37-44.

242	da Silva, I. A. A., et al. (2014). Fragmentation and loss of habitat: consequences for the fern communities in Atlantic forest remnants in Alagoas, north-eastern Brazil. Plant Ecology & Diversity 7(4): 509-517.
243	De Beenhouwer, M. (2011). Effects of habitat fragmentation and coffee cultivation on the diversity of epiphytic orchids in Ethiopian Afromontane forests. Leuven, NL, K. U. Leuven. Master in Biology: 1-59.
244	Decker, M., et al. (2011). Composition and diversity of epiphytes and birds in different types and densities of dispersed trees in silvopastoral systems in the Copán river sub-basin, Honduras. Agroforestería en las Américas 48: 46-53.
245	Dunn, R. R. (2000). Bromeliad communities in isolated trees and three successional stages of an Andean cloud forest in Ecuador. Selbyana 21: 137-143.
246	Einzmann, H. J. R., et al. (2016). Epiphytes in human settlements in rural Panama. Plant Ecology & Diversity 9(3): 277-287.
247	Einzmann, H. J. R. and G. Zotz (2016). How diverse are epiphyte assemblages in plantations and secondary forests in tropical lowlands? Tropical Conservation Science 9(2): 629-647.
248	Elias, J. P. C., et al. (2021). Host tree traits in pasture areas affect forest and pasture specialist epiphyte species differently. American Journal of Botany 108(4): 598-606.
249	Fernandes Schievenin, D., et al. (2024). Traits of the host trees, not community diversity, drive epiphytes abundance in tropical seasonal forests. Biota Neotropica 24: e20231558.
250	Flores-Argüelles, A., et al. (2022). Diversity and vertical distribution of epiphytic angiosperms, in natural and disturbed forest on the northern coast of Jalisco, Mexico. Frontiers in Forests and Global Change 5: 828851.
251	Flores-Palacios, A. and J. G. Garcia-Franco (2004). Effect of isolation on the structure and nutrient content of oak epiphyte communities. Plant Ecology 173(2): 259-269.
252	Flores-Palacios, A. and J. G. Garcia-Franco (2008). Habitat isolation changes the beta diversity of the vascular epiphyte community in lower montane forest, Veracruz, Mexico. Biodiversity and Conservation 17(1): 191-207.
253	Gonçalves, L. J. B., et al. (2020). The palm *Syagrus coronata* proliferates and structures vascular epiphyte assemblages in a human-modified landscape of the Caatinga dry forest. Journal of Tropical Ecology 36(3): 123-132.
254	Gradstein, S. R. (2008). Epiphytes of tropical montane forests-impact of deforestation and climate change. The Tropical Mountain Forest - Patterns and Processes in a Biodiversity Hotspot. S. Gradstein, Robbert, J. Homeier and D. Gansert. Göttingen, Germany, Göttingen Centre for Biodiversity. 2: 51-65.
255	Haro-Carrión, X., et al. (2009). Conservation of vascular epiphyte diversity in shade cacao plantations in the Chocó region of Ecuador. Biotropica 41(4): 520-529.
256	Hernández-Pérez, E. and E. Solano (2015). Effects of habitat fragmentation on the diversity of epiphytic orchids from a montane forest of southern Mexico. Journal of Tropical Ecology 31(2): 103-115.

257	Hernández Rodríguez, D. E. (2022). Effect of intensity of high Andean forest use on epiphytic orchid assemblage. Master's thesis. Colombia.
258	Hietz, P. (1999). Diversity and conservation of epiphytes in a changing environment. Pure and Applied Chemistry 70(11): 1-11.
259	Hietz, P., et al. (2006). Effect of forest disturbance on abundance and distribution of epiphytic bromeliads and orchids. Ecotropica 12(2): 103-112.
260	Hietz-Seifert, U., et al. (1996). Epiphyte vegetation and diversity on remnant trees after forest clearance in southern Veracruz, Mexico. Biological Conservation 75(2): 103-111.
261	Higuera, D. and J. H. D. Wolf (2010). Vascular epiphytes in dry oak forests show resilience to anthropogenic disturbance, Cordillera Oriental, Colombia. Caldasia 32(1): 161-174.
262	Köster, N., et al. (2009). Conservation of epiphyte diversity in an Andean landscape transformed by human land use. Conservation Biology 23(4): 911-919.
263	Köster, N., et al. (2011). Effect of host tree traits on epiphyte diversity in natural and anthropogenic habitats in Ecuador. Biotropica 43(6): 685-694.
264	Krömer, T., et al. (2014). Vascular epiphytes as bioindicators of forest quality: anthropogenic impact on their diversity and composition. Bioindicadores guardianes de nuestro futuro ambiental. Z. González, C.A, A. Vallarino, J. C. Pérez-Jimenez and A. M. Low-Pfeng. México, D. F. and Campeche, Mexico, Instituto Nacional de Ecología y Cambio Climático (INECC) – El Colegio de la Frontera Sur (ECOSUR): 605-623.
265	Krömer, T., et al. (2021). Anthropic effects on the diversity of vascular epiphytes and orchids in central Veracruz. Las orquídeas de Veracruz. J. Viccon-Esquivel, M. Castañeda-Zárate, R. Castro-Cortés and W. Cetzal. Xalapa, Mexico, Universidad Veracruzana: 235-252.
266	Larrea, M. L. and F. A. Werner (2010). Response of vascular epiphyte diversity to different land-use intensities in a neotropical montane wet forest. Forest Ecology and Management 260(11): 1950-1955.
267	Moorhead, L. C., et al. (2010). Epiphyte biodiversity in the coffee agricultural matrix: canopy stratification and distance from forest fragments. Conservation Biology 24(3): 737-746.
268	Nkongmeneck, B.-A., et al. (2002). Epiphyte diversity in primary and fragmented forests of Cameroon, Central Africa: a preliminary survey. Selbyana 23(1): 121-130.
269	Oswaldo, J., et al. (2022). Successional forests stages influence the composition and diversity of vascular epiphytes communities from Andean Montane Forests. Ecological Indicators 143: 109366.
270	Page, N. V., et al. (2010). Plant diversity in sacred forest fragments of Western Ghats: a comparative study of four life forms. Plant Ecology 206(2): 237-250.
271	Parra-Tabla, V., et al. (2011). Population status and reproductive success of an endangered epiphytic orchid in a fragmented landscape. Biotropica 43(5): 640-647.

272	Pereira, A. F. D., et al. (2014). Edge effects on fern community in an Atlantic Forest remnant (Bonito, Pernambuco, Brazil). Interciencia 39(4): 281-287.
273	Prevedello, J. A., et al. (2018). The importance of scattered trees for biodiversity conservation: A global meta-analysis. Journal of Applied Ecology 55(1): 205-214.
274	Reid, J. L., et al. (2016). Tropical forest restoration enriches vascular epiphyte recovery. Applied Vegetation Science 19(3): 508-517.
275	Rodríguez Quiel, C. and G. Zotz (2021). Vascular epiphyte assemblages on isolated trees along an elevational gradient in Southwest Panama. Diversity 13(2): 49.
276	Siaz Torres, S. S., et al. (2024). Species richness, abundance, and vertical distribution of epiphytic bromeliads in primary forest and disturbed forest. Plants 13: 2754.
277	Toledo-Aceves, T., et al. (2014). Significance of remnant cloud forest fragments as reservoirs of tree and epiphytic bromeliad diversity. Tropical Conservation Science 7(2): 240-253.
278	Wang, Q., et al. (2017). Tree size predicts vascular epiphytic richness of traditional cultivated tea plantations in Southwestern China. Global Ecology and Conservation 10: 147-153.
279	Werner, F. A. and S. R. Gradstein (2008). Seedling establishment of vascular epiphytes on isolated and enclosed forest trees in an Andean landscape, Ecuador. Biodiversity and Conservation 17(13): 3195-3207.
280	Werner, F. A. and S. R. Gradstein (2009). Diversity of dry forest epiphytes along a gradient of human disturbance in the tropical Andes. Journal of Vegetation Science 20(1): 59-68.
281	Werner, F. A. and S. R. Gradstein (2010). Spatial distribution and abundance of epiphytes along a gradient of human disturbance in an Interandean dry valley, Ecuador. Selbyana 30(2): 208-215.
282	Werner, F. A., et al. (2005). Diversity of vascular epiphytes on isolated remnant trees in the montane forest belt of southern Ecuador. Ecotropica 11: 21-40.
283	Werner, F. A., et al. (2011). Is the resilience of epiphyte assemblages to human disturbance a function of local climate? Ecotropica 17(2): 15-20.
284	Wolf, J. H. D. (2005). The response of epiphytes to anthropogenic disturbance of pine-oak forests in the highlands of Chiapas, Mexico. Forest Ecology and Management 212(1-3): 376-393.
285	Woods, C. L. and S. J. DeWalt (2013). The conservation value of secondary forests for vascular epiphytes in Central Panama. Biotropica 45(1): 119-127.

Table S2. Comparison of the frequency of words in the different clusters (A, B, C, D), the frequency indicates the number of times the a word is repeated in a given cluster, it ranges from 1 (singletons) to 1298, whereby some cluster have a large number of common words (A and D) and other a large number of “rare” words or singletons (B and C); the differences in the abundance structure of the words per cluster can be contrasted graphically with Figure S3.

**Frequency**	**A**	**B**	**C**	**D**
1	0	43	1853	0
2	0	13	571	0
3	0	18	261	0
4	0	14	203	0
5	0	9	109	0
6	1	8	106	0
7	1	11	65	0
8	0	11	62	0
9	2	7	42	0

10	3	6	39	0
11	3	7	31	0
12	0	12	18	0
13	3	5	16	0
14	6	4	24	0
15	0	8	14	0
16	2	12	21	0
17	2	3	12	0
18	2	9	12	1
19	2	8	9	0
20	1	4	11	0
21	1	6	4	0

22	2	5	5	0
23	1	2	7	0
24	1	6	8	0
25	2	4	4	0
26	1	2	3	0
27	1	6	2	0
28	1	5	7	1
29	0	4	3	2
30	3	3	3	1
31	1	2	4	0
32	0	0	3	1
33	2	4	3	0

34	4	2	3	0
35	4	3	0	0
36	1	3	3	1
37	0	2	3	0
38	1	2	2	1
39	1	2	0	0
40	3	1	1	2
41	1	3	1	0
42	3	5	3	0
43	0	4	3	1
44	2	3	1	0
45	1	0	2	1

46	1	4	0	0
47	0	1	1	1
48	0	2	0	0
49	1	1	1	2
50	0	1	1	1
51	0	2	0	0
52	0	3	1	1
53	1	2	0	1
54	0	1	0	0
55	0	1	1	0
56	1	2	0	0
57	2	2	0	0

58	0	2	0	0
59	0	1	2	1
60	0	2	0	0
61	0	3	0	1
62	1	3	2	1
63	0	1	0	1
64	0	1	0	0
65	2	1	0	0
66	2	0	0	0
67	0	1	1	1
68	0	1	0	0
69	0	1	0	1

71	1	4	0	0
72	0	0	1	0
73	1	1	0	0
75	0	1	0	1
76	0	1	0	2
78	0	0	0	1
79	1	1	0	0
81	0	0	0	1
83	1	0	0	0
86	0	2	0	0
87	0	1	0	0
88	0	1	0	1

90	0	1	0	0
93	0	0	0	1
95	0	0	0	1
97	1	1	0	1
98	0	0	0	1
101	0	0	0	1
102	0	0	1	0
107	0	0	0	1
109	2	1	0	0
110	0	0	0	1
111	1	0	0	1
112	0	0	0	1

114	0	0	0	1
116	0	0	1	0
119	0	1	0	0
122	0	1	0	0
123	0	0	0	1
124	0	0	1	0
128	0	1	0	0
130	1	0	1	0
136	0	1	0	0
137	0	0	0	1
138	0	0	1	1
139	1	0	0	0

143	0	0	1	0
145	0	0	0	1
146	0	0	0	1
148	0	1	0	0
149	0	0	0	1
154	0	0	0	1
156	0	0	0	2
182	1	1	0	0
189	1	0	0	0
191	0	0	0	1
208	0	0	0	1
210	1	0	0	0

213	0	1	0	0
222	0	0	0	1
225	0	0	0	1
227	0	0	0	1
252	0	0	0	1
256	0	0	0	1
270	0	0	0	1
276	0	0	0	1
310	0	0	0	1
325	1	0	0	0
353	1	0	0	0
419	0	0	0	1

898	0	0	0	1
1167	0	0	1	0
1298	0	0	0	1

Table S3. Comparison of epiphyte species-richness reduction in old-growth vs. secondary forest fragments (SFF) at different study regions, indicating vegetation type (VT), elevational range (ER), annual precipitation (AP), and references in chronological order.

**Study region and country**	**VT**	**ER (m asl)**	**AP (mm)**	**Age of SFF (years)**	**Reduction (%) (species-richness comparison)**	**Forest types compared**	**Reference**
Venezuelan Andes, north-west of Mérida	Humid montane forest	2200-2700	1460	23	-96 (191 : 7 spp.)	Mosaic of primary forest fragments and secondary forests	Barthlott et al. (2001)
Bolivian Andes, Yungas of La Paz	Humid sub-montane and montane forest	500-625 and 1600-1900	1500-2000	15<br>15	-61 (114 : 44 spp.)<br>-73 (160.5 : 43 spp.)	Natural forests vs. neighbouring fallows at two sites with different elevations	Krömer and Gradstein (2003)
Colombian Amazon, Amacayacu National Park	Lowland rain forest	80-200	3200	9-16<br>17-22<br>23-30	-66 (15.4 : 5.3 spp.)<br>-46 (15.4 : 8.2 spp.)<br>27 (15.4 : 11.3 spp.)	Mature vs. secondary forests	Benavides et al. (2006)
Ecuadorian Andes, Otonga Reserve	Humid montane forest	1650-2250	2584	10<br>> 24	-63 (84 : 30.8 spp.)<br>-22 (84 : 65.8 spp.)	Mosaic of primary forest fragments and secondary forests	Köster et al. (2009)
Ecuadorian Andes, Bosque Protector Jerusalén in the Guayllabamba drainage north of Quito	Inter-Andean dry forest	2300-2320	530	13-28<br>20	-14 (3.5 : 3.0 spp.)<br>-6 (3.5 : 3.3 spp.)	Closed-canopy mixed forest vs. young secondary forests	Werner and Gradstein (2009)

Western Mexico, pacific coast, Reserva de la Biosfera Chamela-Cuixmala	Lowland dry forest	0-500	400-1300	5-8<br><br><br>10-20	61.5%<br>(2.6 : 1.0 spp.)<br>42%<br>(2.6 : 1.5 spp.)	Trees in mature forest vs. young secondary forests	Cuevas-Reyes and Gutiérrez (2012)
Central Panama, Barro Colorado Nature Monument	Lowland rain forest	120-160	2600	35<br>55<br>85<br>115	-86 (7 : 1 spp.)<br>-79 (7 : 1.5 spp.)<br>-64 (7: 2.5 spp.)<br>-29 (5 : 7 spp.)	Old-growth vs. secondary forest	Woods and DeWalt (2013)
Eastern Mexico, gulf coast, Los Tuxtlas Biosphere Reserve	Lowland rain forest	35-560	3000	20	-29 (59 : 42 spp.)	Old-growth forest vs. nearby secondary forest	Pérez-Peña and Krömer (2017)
Argentina, province of Tucuman, Parque Sierra de San Javier	Subtropical montane forest	700-1000	1200-1300	30<br>60	-27 (22 : 16 spp.)<br>-4.5 (22 : 21 spp.)	Mature forests vs. secondary forests	Ceballos (2020)
Eastern Mexico, Sierra Madre Oriental, central Veracruz	Humid montane forest	1250-1670	1650-2270	15-25	-58 (90 : 38 spp.)	Old-growth forest vs. adjacent secondary forest	Krömer et al. (2021)

Table S4. Comparison of epiphyte species-richness reduction in old-growth forest vs. different types of plantations at different study regions, indicating vegetation type (VT), elevational range (ER), annual precipitation (AP), and references in chronological order.

**Study region and country**	**VT**	**ER (m asl)**	**AP (mm)**	**Type of plantation**	**Reduction (%) (species-richness comparison)**	**Forest types compared**	**Reference**
Eastern Mexico, Sierra Madre Oriental, central Veracruz	Humid montane forest	1250-1410	1500-2000	Coffee plantation with *Inga* and *Citrus* as shade trees	Orchids only<br>-43 (44 : 25 spp.)	Old-growth forest vs. shaded coffee plantation	Williams-Linera et al. (1995)
Eastern Mexico, gulf coast, Los Tuxtlas Biosphere Reserve	Lowland rain forest	100-250	3000	*Cedrela odorata* and *Citrus* spp. cultivated in pasture	-57 (35 : 15 spp.)	Old-growth forest vs. cultivated citrus and *Cedrela* trees	Hietz-Seifert et al. (1996)
Eastern Mexico, Sierra Madre Oriental, central Veracruz	Tropical oak forest or semievergreen tropical forest	720	1200	Mango plantation	-74 (19 : 5 spp.)	Old-growth forest trees vs. old mango trees	Hietz (1998)
Venezuelan Andes, north-west of Mérida, La Carbonera	Humid montane forest	2200-2700	1460	32 year-old plantation of *Cedrela montana*	-93 (178 : 13 spp.)	Mosaic of primary forest fragments and a tree plantation	Barthlott et al. (2001)
Eastern Mexico, Sierra Madre Oriental, central Veracruz	Humid montane forest in transition to semievergreen tropical forest	720-1100	1200-1300	Traditional coffee polycultures with old shade trees and commercial monoculture with small shade trees	-28 (mean: 44.5 : 32 spp.)<br>67 (mean: 44.5 : 14.5 spp.)	Trees in four old-growth forest sites vs. shade trees in eight coffee plantations	Hietz (2005)

Northwestern Ecuador, Esmeraldas Province in Chocó region	Lowland rain forest	200	3000	Relict trees in rustic shade cacao plantations	-30 (77 : 54 spp.)	Large trees in old-growth forests vs. adjacent rustic shade cacao plantations	Haro-Carrión et al. (2009)
Southern Mexico, Soconusco region in Chiapas State	Humid montane forest	800-1200	4500	Traditional coffee polyculture with high density of shade trees and commercial monoculture with low density	-24 (58 : 44 spp.)<br>-55 (58 : 26 spp.)	Old-growth forest vs. shaded coffee plantations	Moorhead et al. (2010)
Brazil, Rio Grande do Sul State, Sao Francisco de Paula National Forest	Humid *Araucaria* Forest	600-950	2252	Plantations of *Araucaria*, *Pinus*, and *Eucalyptus*	-52 (54 : 26 spp.)<br>-85 (54 : 8 spp.)<br>-89 (54 : 6 spp.)	Araucaria Forest vs. plantations of Araucaria, Pinus, and Eucalyptus	Boelter et al. (2011)
Eastern Mexico, Sierra Madre Oriental, central Veracruz	Humid montane forest	1000-1400	1500-2000	Traditional coffee polycultures with old shade trees and *Pinus patula* plantation	Ferns only<br>-45.5 (22 : 12 spp.)<br>-91 (22 : 2 spp.)	Old-growth forest vs. shaded coffee plantation and pine plantation	Carvajal-Hernández et al. (2014)
Southwestern Ethiopia, Manna and Gera districts of the Jimma region	Moist evergreen afromontane forest	1870-2085	1800-2300	Shade trees in large and small managed semiforest coffee systems	Orchids only<br>-30 (20 : 14 spp.)<br>-55 (20 : 9 spp.)	Canopy trees in old-growth unfragmented forest vs. intensively managed coffee agroecosystems	Hundera et al. (2015)
East of central Sumatra, Indonesia,	Lowland rain forest	0-500	2235	Rubber and oil palm plantations	-74 (43 : 11 spp.)<br>-79 (43 : 9 spp.)	30 plots of 20 × 20 m each in primary rainforest	Böhnert et al. (2016)

Bukit Duabelas National Park in Jambi						vs. rubber and oil palm plantations	
Panama, Pacific slope, provinces of Chiriquí, Veraguas, Herrera and Los Santos	Lowland rain forest	< 500	1100-4200	Teak, oil palm and pine plantations	-59 (46 : 19 spp.)<br>-89 (46 : 5 spp.)<br>-96 (46 : 2 spp.)	Secondary forest patches vs. three types of plantations	Einzmann and Zotz (2016)
Eastern Mexico, gulf coast, Los Tuxtlas Biosphere Reserve	Lowland rain forest	35-560	3000	Old and abandoned citrus plantation	-14 (59 : 51 spp.)	Old-growth forest vs. nearby citrus plantation	Pérez-Peña and Krömer (2017)
Eastern Mexico, Sierra Madrigal in Tabasco State	Lowland rain forest	60-500	3600	Shaded cocoa plantations	Orchids only<br>-31 (32 : 22 spp.)	Old-growth forests vs. adjacent shaded cacao plantations	Morales-Linares et al. (2020)
North-central Nicaragua, province of Jinotega	Humid montane forest	1000-1300	1660	Traditional coffee polycultures with old shade trees	-12.5 (80 : 70 spp.)<br>-24 (62 : 47 spp.)	Trees in preserved forest vs. shade trees in two coffee farms	Richards et al. (2020)
Eastern Mexico, Sierra Madre Oriental, central Veracruz	Humid montane forest	1000-1670	1650-2500	Commercial coffee plantation with sparse old shade trees	-53 (90 : 43 spp.)	Old-growth forest vs. shaded coffee plantation	Krömer et al. (2021)
Southwestern Ethiopia, Kafa biosphere reserve in Gimbo district	Moist evergreen afromontane forests	500-3300	1710-1892	Remnant shade trees in the coffee agroecosystem	-67.5 (40 : 13)	Forest trees vs. nearby coffee agroecosystems	Osie et al. (2022)

Table S5. Comparison of epiphyte species-richness reduction in old-growth forest vs. isolated remnant trees in pastures at different study regions, indicating vegetation type (VT), elevational range (ER), annual precipitation (AP), and references in chronological order.

**Study region and country**	**VT**	**ER (m asl)**	**AP (mm)**	**Type of remnant trees**	**Reduction (%)**	**Forest types compared**	**Reference**
Eastern Mexico, Sierra Madre Oriental, central Veracruz	Humid montane forest	1250-1410	1500-2000	Isolated trees within pasture	Orchids only -21.5 (44 : 35 spp.)	Old-growth forest vs. pasture with remnant trees	Williams-Linera et al. (1995)
Eastern Mexico, gulf coast, Los Tuxtlas Biosphere Reserve	Lowland rain forest	100-250	3000	38 isolated remnant forest trees in a pasture established some 28 years ago	+66 (35 : 58 spp.)	Old-growth forest vs. pasture with remnant trees	Hietz-Seifert et al. (1996)
Southern Ecuador, Cordillera El Consuelo, valley of the Río San Francisco, near the Estación Científica San Francisco	Humid montane forest	1800-2200	2000	Canopy trees in primary forest and isolated remnant trees	-70 (225 : 67 spp.)	Six forest trees vs. 15 isolated remnant trees of pastures	Werner et al. (2005)
Eastern Mexico, Sierra Madre Oriental, central Veracruz	Humid montane forest	1500-1600	1650	Isolated pastureland trees	-26 (70 : 52 spp.)	Two old-growth forest plots vs.	Flores-Palacios and García-Franco (2008)

						pasture with remnant trees	
Ecuadorian Andes, Otonga Reserve	Humid montane forest	1650-2250	2584	Isolated trees within pasture	-17 (286 : 237 spp.)	Mosaic of primary forest fragments vs. pasture with remnant trees	Köster et al. (2009)
Ecuadorian Andes, Bosque Protector Jerusalén in the Guayllabamba drainage north of Quito	Inter-Andean dry forest	2300-2320	530	Isolated trees in grassland	-43 (21 : 12 spp.)	Closed-canopy mixed forest vs. isolated trees in grassland	Werner and Gradstein (2009)
Northeastern Ecuador, between the Cordilleras Oriental and Cordillera de los Huacamayos, private reserve of Sierrazul, Napo Province	Humid montane forest	2250	3000-3500	Isolated remnant trees in cattle pastures	-8 (115 : 106 spp.)	Unmanaged mature forest vs. isolated remnant trees in cattle pastures 6 years following forest clearance	Larrea and Werner (2010)
Southwest Panama in the province of Chiriquí	Lowland rain forest	< 200	2500-3500	Isolated pasture trees	-35 (68 : 44 spp.)	Forest plots vs. pasture plots	Poltz and Zotz (2011)
Eastern Mexico, Sierra Madre Oriental, central Veracruz	Humid montane forest	1000-1400	1500-2000	Pastizal inducido con árboles remanentes en un área de 15 ha	Ferns only -68 (22 : 7 spp.)	Old-growth forest vs. pasture with remnant trees	Carvajal-Hernández et al. (2014)

Southeastern Mexico, Depresión Central de Chiapas	Tropical dry forest	790-1020	800	Remnant *Ficus* trees in pastures	-64 (45 : 16 spp.)	Sampling of vascular epiphytes on *Ficus* trees in silvo-pastoral systems and semi-conserved tropical deciduous forest	Trejo-Cruz et al. (2021)
Southern Brazil, Santa Virginia Nucleus of Serra do Mar State Park, municipalities of Taubaté, São Luiz do Paraitinga, and Lagoinha, São Paulo State	Atlantic montane forest	860-1470	1400-2000	Isolated pasture trees	-99 (172 : 2 spp.)	Canopy trees in old-growth forest vs. isolated trees in pastures	Parra-Sanchez and Banks-Leite (2022)

Table S6. Comparison of epiphyte species-richness reduction in old-growth forest vs. disturbed, managed or selectively logged forests at different study regions, indicating vegetation type (VT), elevational range (ER), annual precipitation (AP), and references in chronological order.

**Study region and country**	**VT**	**ER (m asl)**	**AP (mm)**	**Type of disturbed forest**	**Reduction (%)**	**Forest types compared**	**Reference**
Venezuelan Andes, north-west of Mérida	Humid montane forest	2200-2700	1460	Relict trees in disturbed forest, where the last selective logging events took place some 50 years ago	-76 (178 : 42 spp.)	Primary forest fragments vs. disturbed forests	Barthlott et al. (2001)
India, foothills of the Eastern Himalaya, Pakhui Wildlife Sanctuary in Arunachal Pradesh State	Moist, semi-evergreen lowland forest	500-850	2500	Selectively logged and unlogged forests with treefall gaps	-29 (41 : 29 spp.)<br>-15 (41 : 35 spp.)	Closed forest vs. selectively logged and unlogged forests with treefall gaps	Padmawathe et al. (2004)
Southern Mexico, Central Plateau, municipality of San Cristóbal de Las Casas in Chiapas State	Pine-oak forest	2160-2490	1042	Selectively logged forests and ca. 25 years old plantation-like oak coppices with regularly clear-cut	-2 (28,3 : 27,7 spp.)<br>-51 (28,3 : 14 spp.)	Three sites each of least disturbed mature forests vs. selectively logged and cyclically clear-cut forests	Wolf (2005)
Eastern Mexico, gulf coast, Veracruz State	*Sabal mexicana* forests	10	1300	Palms and trees in perturbed sites (managed pastures)	+64 (11 : 18 spp.)	30 pairs of phorophytes consisting of an *S. mexicana* palm and a	Aguirre et al. (2010)

						nonpalm tree in conserved and perturbed sites	
Northeastern Ecuador, between the Cordilleras Oriental and Cordillera de los Huacamayos, private reserve of Sierrazul, Napo Province	Humid montane forest	2250	3000-3500	Managed forest fragment with mid- and understorey opened for cattle grazing	-10.5 (115 : 103 spp.)	Unmanaged mature forest vs. managed forest	Larrea and Werner (2010)
Eastern Mexico, Sierra Madre Oriental, central Veracruz	Humid montane forest	1000-1670	1650-2500	Fragment of forest disturbed by extraction of firewood, lumber and non-timber products	-49 (90 : 46 spp.)	Old-growth forest vs. disturbed forest	Krömer et al. (2021)
India, southern Western Ghats, Agasthyamalai Biosphere Reserve	Wet-evergreen montane forests	1300-1400	3000-3500	Historically selectively logged forest (40 years before)	-11 (18 : 16 spp.)	100 trees each in an unlogged and a selectively logged forest	Seshadri et al. (2021)
Western Mexico, municipality of Cabo Corrientes in the western part of Jalisco State	Tropical deciduous and semideciduous forests	0-300	1625	Mature trees in three different disturbed forest types	+28 (23 : 18 spp.) +18 (13 : 11spp.) +90 (19 : 10 spp.)	Old-growth and disturbed sites in three types of vegetation (gallery forest, oak forest, tropical semideciduous forest)	Flores-Argüelles et al. (2022)
Southern Mexico, Sierra Madre of Chiapas, Los	Pine-oak forest	800-1500	1250	Two managed forests subjected to thinning and release cutting	-15 (52 : 44 spp.)	Unharvested old-secondary forest vs. two managed forest stands	Martínez-Meléndez et al. (2022)

Ocotones, municipality of Cintalapa de Figueroa					-25 (52: 39 spp.)		
Southern Brazil, Santa Virginia Nucleus of Serra do Mar State Park, municipalities of Taubaté, São Luiz do Paraitinga, and Lagoinha, São Paulo State	Atlantic montane forest	860-1470	1400-2000	Human-modified forests surrounded by pastures	-84 (172 : 28 spp.)	Old-growth continuous forest vs. human-modified forests	Parra-Sanchez and Banks-Leite (2022)
